# Atacama Large Aperture Submillimeter Telescope (AtLAST) science: Surveying the distant Universe

**DOI:** 10.12688/openreseurope.17445.1

**Published:** 2024-06-24

**Authors:** Eelco van Kampen, Tom Bakx, Carlos De Breuck, Chian-Chou Chen, Helmut Dannerbauer, Benjamin Magnelli, Francisco Miguel Montenegro-Montes, Teppei Okumura, Sy-Yin Pu, Matus Rybak, Amelie Saintonge, Claudia Cicone, Evanthia Hatziminaoglou, Juliëtte Hilhorst, Pamela Klaassen, Minju Lee, Christopher C. Lovell, Andreas Lundgren, Luca Di Mascolo, Tony Mroczkowski, Laura Sommovigo, Mark Booth, Martin A. Cordiner, Rob Ivison, Doug Johnstone, Daizhong Liu, Thomas J. Maccarone, Matthew Smith, Alexander E. Thelen, Sven Wedemeyer

**Affiliations:** 1 European Southern Observatory, Garching bei München, Bayern, 85748, Germany; 2 Department of Space, Earth, & Environment, Chalmers University of Technology, Gothenberg, SE-412 96, Sweden; 3 Academia Sinica Institute of Astronomy and Astrophysics, Taipei, 10617, Taiwan; 4 Instituto de Astrofisica de Canarias, La Laguna, Tenerife, E-38205, Spain; 5 Dpto. Astrofisica, Universidad de La Laguna, La Laguna, E-38206, Spain; 6 CEA, CNRS, AIM, Université Paris-Saclay, Université Paris-Cité, Gif-sur-Yvette, 91191, France; 7 Departamento de Física de la Tierra y Astrofísica e Instituto de Fisica de Particulas y del Cosmos (IPARCOS), Universidad Complutense de Madrid, Madrid, 28040, Spain; 8 Graduate Institute of Astronomy, National Tsing Hua University, Hsinchu, 30013, Taiwan; 9 Faculty of Electrical Engineering, Mathematics and Computer Science, Delft University of Technology, Delft, 2628 CD, The Netherlands; 10 Leiden Observatory, Leiden University, Leiden, 2333 CA, The Netherlands; 11 SRON - Netherlands Institute for Space Research, Leiden, 2333 CA, The Netherlands; 12 Department of Physics and Astronomy, University College London, London, WC1E 6BT, UK; 13 Max-Planck-Institut für Radioastronomie, Bonn, D-53121, Germany; 14 Institute of Theoretical Astrophysics, University of Oslo, Oslo, N-0315, Norway; 15 Department of Astronomy, Yale University, New Haven, Connecticut, 06511, USA; 16 UK Astronomy Technology Centre, Royal Observatory Edinburgh, Edinburgh, EH9 3HJ, UK; 17 Cosmic Dawn Centre, Copenhagen, Denmark; 18 DTU-Space, Technical University of Denmark, Kgs. Lyngby, DK 2800, Denmark; 19 Institute of Cosmology and Gravitation, University of Portsmouth, Portsmouth, PO1 3FX, UK; 20 CNRS, CNES, LAM, Aix Marseille Univ, Marseille, France; 21 Laboratoire Lagrange, Observatoire de la Côte d'Azur, CNRS, Universite Cote d'Azur, Nice, 06304, France; 22 Astronomy Unit, Department of Physics, University of Trieste, Trieste, 34131, Italy; 23 INAF - Osservatorio Astronomico di Trieste, Trieste, 34131, Italy; 24 IFPU - Institute for Fundamental Physics of the Universe, Trieste, 34014, Italy; 25 Center for Computational Astrophysics, Flatiron Institute, New York, New York, 10010, USA; 26 Astrochemistry Laboratory, NASA Goddard Space Flight Center, Greenbelt, MD, 20771, USA; 27 School of Cosmic Physics, Dublin Institute for Advanced Studies, Dublin, D02 XF86, Ireland; 28 Institute for Astronomy, University of Edinburgh, Edinburgh, EH9 3HJ, UK; 29 ARC Centre of Excellence for All Sky Astrophysics in 3 Dimensions, Canberra, Australia; 30 NRC Herzberg Astronomy and Astrophysics, Victoria, BC, V9E 2E7, Canada; 31 Department of Physics and Astronomy, University of Victoria, Victoria, British Columbia, V8P 5C2, Canada; 32 Max-Planck-Institut für extraterrestrische Physik, Garching bei München, Bayern, D-85748, Germany; 33 Purple Mountain Observatory, Chinese Academy of Sciences, Nanjing, 210023, China; 34 Department of Physics & Astronomy, Texas Tech University, Lubbock, Texas, 79409-1051, USA; 35 School of Physics & Astronomy, Cardiff University, Cardiff, CF24 3AA, UK; 36 Division of Geological and Planetary Sciences, California Institute of Technology, Pasadena, California, CA 91125, USA; 37 Rosseland Centre for Solar Physics, University of Oslo, Oslo, N-0315, Norway

**Keywords:** cosmology, galaxy surveys, galaxy formation, sub-mm galaxies, cluster galaxies

## Abstract

During the most active period of star formation in galaxies, which occurs in the redshift range 1 *< z <* 3, strong bursts of star formation result in significant quantities of dust, which obscures new stars being formed as their UV/optical light is absorbed and then re-emitted in the infrared, which redshifts into the mm/sub-mm bands for these early times. To get a complete picture of the high- *z* galaxy population, we need to survey a large patch of the sky in the sub-mm with sufficient angular resolution to resolve all galaxies, but we also need the depth to fully sample their cosmic evolution, and therefore obtain their redshifts using direct mm spectroscopy with a very wide frequency coverage.

This requires a large single-dish sub-mm telescope with fast mapping speeds at high sensitivity and angular resolution, a large bandwidth with good spectral resolution and multiplex spectroscopic capabilities. The proposed 50-m Atacama Large Aperture Submillimeter Telescope (AtLAST) will deliver these specifications. We discuss how AtLAST allows us to study the whole population of high-z galaxies, including the dusty star-forming ones which can only be detected and studied in the sub-mm, and obtain a wealth of information for each of these up to *z ∼* 7: gas content, cooling budget, star formation rate, dust mass, and dust temperature.

We present worked examples of surveys that AtLAST can perform, both deep and wide, and also focused on galaxies in proto-clusters. In addition we show how such surveys with AtLAST can measure the growth rate f *σ*_8_ and the Hubble constant with high accuracy, and demonstrate the power of the line-intensity mapping method in the mm/sub-mm wavebands to constrain the cosmic expansion history at high redshifts, as good examples of what can uniquely be done by AtLAST in this research field.

## Plain language summary

Galaxies come in a wide variety of shapes, sizes, and colours, despite all of them having originated from similar initial conditions in the early Universe. Understanding this diversity by tracing back the evolutionary pathways of different types of galaxies is a major endeavour in modern astrophysics. Galaxies build their stellar mass over time by converting gas into stars through various episodes of star formation. Understanding exactly when, where, and how this star formation process is triggered or suppressed is therefore a crucial question to answer.

Current observations reveal that the Universe was at its most active (in terms of star formation rate per unit volume) in the distant past, about 10 billion years ago. By measuring the amount of gas and dust in galaxies at that epoch, we also know that the reason for this very high star formation activity is large reservoirs of gas (the fuel for star formation) and the higher efficiency of galaxies at converting their gas into stars. However, recent work also reveals that we are missing significant numbers of distant actively star-forming galaxies in current samples because these are obscured by dust, and therefore our picture is still very incomplete.

In this paper, we explore how a new proposed telescope, the Atacama Large Aperture Submillimeter Telescope (AtLAST: http://atlast-telescope.org), can provide us with the very important missing pieces of this puzzle. AtLAST will allow us to map large areas of the sky at unprecedented depth, resolution and multiplex spectroscopic capabilities. This telecope would provide us with a complete, homogeneous and unbiased picture of the star-forming galaxy population in the early Universe. Not only will we be able to discover these galaxies, but also measure their distances, the composition of their gas and dust content, and the rate at which they convert gas into stars.

## 1. Introduction

In the distant Universe, the star formation rate density in galaxies is highest in the redshift range 1 *< z <* 3 (eg. Madau & Dickinson, 2014), which results in a fair amount of astrophysical dust and gas in these galaxies. A similar trend is observed in the cold-gas content of galaxies - as traced by emission lines and cold dust, which is seen to peak at a similar redshift range (e.g. Tacconi *et al.*, 2020). The dust obscures new stars being formed, because their UV/optical light is absorbed and then re-emitted in the infrared (IR, eg. Salim & Narayanan, 2020 for an excellent review on the physical mechanisms), contributing to the so-called cosmic far-IR background (CIB). This accounts for about half of the energy density from star formation, integrated over the history of the Universe ( Dole *et al.*, 2006). The infrared photons emitted in the rest-frame of these galaxies get redshifted into the submillimeter and millimeter ((sub-)mm) observed frame, and the negative K-correction at this wavelength regime enables galaxies to appear roughly constant in the observed flux densities at *z ∼* 1−10 ( Blain *et al.*, 2002). This means that the (sub-)mm is an essential wavelength range for studying high- *z* galaxies in order to understand their star formation and growth. Since high- *z* star forming galaxies are best observed in the (sub-)mm, they are often called ’sub-mm galaxies’ (SMGs), although lately the physically-motivated denomination of ‘dusty star forming galaxies’ (DSFGs) is preferred. The study of DSFGs now forms a rich research field: for reviews on its history see Carilli & Walter (2013), Casey *et al.* (2014), Combes (2018). In the 25 years since the discovery of DSFGs a number of surveys have targeted the (dust) continuum and spectral line emission from these high-redshift galaxies.

Wide-field deep continuum surveys with many-pixel bolometer detectors on single-dish telescopes have been efficient in discovering DSFGs out to the epoch of reionisation ( *z ≈* 6). From the ground, the South Pole Telescope (SPT) conducted a *∼*2500 deg ^2^ shallow survey at 1.4 mm and 2 mm, uncovering almost a hundred (mostly gravitationally lensed) dusty galaxies ( Everett *et al.*, 2020; Reuter *et al.*, 2020 and references therein). A similar survey has been completed by the 6-m Atacama Cosmology Telescope (ACT) ( Gralla *et al.*, 2020). At 850 *µ*m, several deg ^2^ have been surveyed by the LABOCA camera on APEX ( Weiß *et al.*, 2009) and the SCUBA-2 camera on JCMT ( Geach *et al.*, 2017). Due to their low angular resolution, these surveys are confusion-limited at the mJy level, i.e., faint sources start overlapping. Current continuum survey facilities include the NIKA-2 camera on the 30-m IRAM telescope, A-MKID 350/850- *µ*m camera on APEX, and the TolTEC camera on the 50-m LMT.

In space, *Herschel* mapped up to 1270 deg ^2^ at 250 - 500 *µ*m, revealing 1.7 million dusty galaxies (with multiple detections), as collected in the *Herschel* Extragalactic Legacy Project (HELP, Shirley *et al.*, 2021), which notably includes the *Herschel* Multi-tiered Extragalactic Survey (HerMES Oliver *et al.*, 2012) and the *Herschel* Atlas survey (H-ATLAS, Eales *et al.*, 2010). About a dozen extremely bright / highly magnified high- *z* galaxies were detected in the all-sky (but relatively shallow) continuum imaging with the *Planck* telescope ( Lammers *et al.*, 2022). The redshift distribution depends on the selection wavelength (e.g. Béthermin *et al.*, 2015), so there is a clear need for a wide frequency range in observations to enable a complete census of dusty galaxies from cosmic noon to cosmic dawn.

ALMA has carried out several “wide-field” continuum surveys, starting with the 1.3-mm survey of the HUDF ( Dunlop *et al.*, 2017, *≈*4.5 arcmin ^2^). The currently most extensive interferometric survey is the 2-mm MORA survey ( Casey *et al.*, 2021, 184 arcmin ^2^), with an extension currently being produced. In contrast to the continuum mapping, blind spectral-line surveys have been limited to interferometers - e.g., ASPECS ( Walter *et al.*, 2016), ALMACAL ( Klitsch *et al.*, 2019), and ALCS ( Fujimoto *et al.*, 2023) on ALMA, and HDF-N survey with NOEMA ( Boogaard *et al.*, 2023, 8 arcmin ^2^). This is because current spectroscopic instruments on single-dish telescopes are often limited to single-pixel designs, and the few exceptions have a maximum of up to ten spatial elements. Instead, the interferometers act as an “integral field unit” (IFU) within their limited field-of-view of interferometers, that cover an area roughly equal to a single pixel element of a single-dish telescope. Multi-object spectroscopy - the ability to obtain spectra of multiple objects in the field-of-view of the telescope, common at optical / near-IR wavelengths - is virtually non-existent in the sub-mm wave-bands. As such, these surveys are inherently restricted to pencil-beam observations due to the low mapping speeds. ALMA Large Programs such as ALPINE ( Béthermin *et al.*, 2020; Faisst *et al.*, 2020) and REBELS ( Bouwens *et al.*, 2022; Inami *et al.*, 2022) have provided the first statistical sample of 4 *< z <* 7 (up to 7) dust continuum emitting galaxies (albeit UV-selected, not DSFGs), but these were targeted surveys, not blind ones. The contribution to the star-formation rate density of these more ’normal’ (but still massive: *M*_∗_ > 10 ^9^, 1 ^10^ *M*_⊙_ for ALPINE/REBELS) dusty galaxies seems to be far from negligible (> 30% at *z*=7, see e.g. Algera *et al.*, 2023; Barrufet *et al.*, 2023). There are many uncertainties due to selection bias, and lack of multiple ALMA observations for most of the targets, but AtLAST will have the sensitivity to detect these sources.

**Figure 1.**
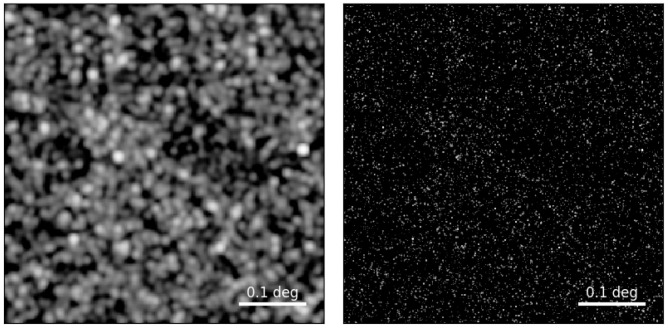
A simulated 277-GHz continuum map with the 6-m ACT telescope (left) and AtLAST (right) *without* instrument noise. The ACT map is limited by confusion noise at X mJy level (notice the seemingly overlapping faint sources); thanks to its 50-m dish, AtLAST will be able to resolve much fainter (up to four orders of magnitude) individual sources. These mock images are based on simulated galaxy catalogues from Lagos *et al.* (2020).

To date, the science driven by sub-mm observations has focused on a combination of large-area continuum observations from both ground- and space-based observations, while spectroscopic observations with a sufficient spatial resolution to resolve these galaxies are limited to small areas. This bimodal approach has left a discovery space that can only be addressed by a telescope with a large primary mirror, exploiting recent advances in instrumentation to provide Integral Field Unit (IFU) capabilities on the scale of individual galaxies. AtLAST is conceived to realise this by combining a large throughput single dish facility with a powerful instrumentation suite. How will AtLAST outperform current facilities? High angular resolution (lowering confusion noise); large collecting area and large focal plane - high survey speed (see e.g. Klaassen *et al.*, 2020; Mroczkowski *et al.*, 2023; Mroczkowski *et al.*, 2024; Ramasawmy *et al.*, 2022). For example, the expected sky area covered by AtLAST with a single pointing will be about 200 times larger than the LMT. In addition, the photometric confusion noise at 350 *µ*m will be a factor of > 10000× lower than that of 6-m telescopes like ACT and CCAT-p ( *<* 0.2 *µ*Jy for AtLAST vs. 2600 *µ*Jy for ACT and CCAT-p). The improvement in angular resolution provided by AtLAST is demonstrated clearly in Figure 1, which compares what can be achieved by current 6-m telescopes like ACT to what AtLAST can do.

Here we describe the compelling science that requires a new facility in order to be achieved - specifically AtLAST, a 50m single dish sub-mm telescope with the capabilities listed above (incl. high mapping efficiency). Such an observatory would enable a large-area spectroscopic survey, expose baryon acoustic oscillations, probe the unresolved power-spectrum of galaxies (i.e., line intensity mapping) and reveal the largest coherent structures in the Universe towards the early Universe.

## 2 Science goals

In the following subsections we outline the high- *z* science goals for AtLAST, focusing on the overall high- *z* galaxy population as well on galaxies in proto-clusters. We also discuss how to extract cosmological parameters from the large survey, and the novel line-intensity mapping technique. We refer to Lee *et al.* (2024) and Di Mascolo *et al.* (2024) for companion AtLAST case studies focused on emission line probes of the cold circumgalactic medium (CGM) of galaxies and on probing the Intra-Cluster Medium (ICM) using the full Sunyaev-Zeldovich (SZ) spectrum to understand the thermal history of the Universe, respectively. Additional AtLAST science cases outside the research fields of the distant Universe and cosmology are presented by Booth *et al.* (2024), Cordiner *et al.* (2024), Klaassen *et al.* (2024), Liu *et al.* (2024), Orlowski-Scherer *et al.* (2024), and Wedemeyer *et al.* (2024). For the purpose of this paper, “high- *z*” refers to *z >* 1.

### 2.1 A large homogeneous galaxy survey in the distant Universe

The integrated spectral energy distribution of the CIB is nowadays relatively well constrained thanks to accumulated observations over the past decades from ground and space facilities, in particular close to the peak of thermal IR emission around 150 *µ*m in the rest-frame. Still, at sub-mm wavelengths, where the contributions from high-redshift galaxies are expected to dominate, a fair fraction of this emission remains unresolved and only the brightest population of DSFGs has been identified and studied in certain detail. Many of those have been identified in blind large area-surveys, their redshift determined thanks to CO spectroscopy in the mm-regime, and then followed up and studied in detail with interferometers like ALMA, as in Reuter *et al.* (2020).

Little is known observationally about the less extreme population of normal dusty galaxies, accounting for the bulk of the objects contributing to the CIB at these wavelengths. Studying this population is crucial to progressing our understanding of numerous open questions like the co-evolution of star formation and black-hole growth, as most high-z star formation occurs in galaxies deeply embedded in dust (see Carraro *et al.*, 2020; Mountrichas & Shankar, 2023). Or the evolution of the dust properties over cosmic time, which is under intense debate (see e.g. Dayal *et al.*, 2022; Di Cesare *et al.*, 2023; Drew & Casey 2022; Hirashita & Il’in, 2022; Sommovigo *et al.*, 2022). Till now, the study of these less extreme sub-mm galaxies has been relying partially on extrapolation of properties of MIR- or radio-selected galaxies with emission in FIR/sub-mm. I Another approach has been studying the physical properties of some of the brightest DSFGs which are associated with lensed systems. Both approaches are severely biased against the brightest end of the population.

In order to determine accurately the number counts and the redshift distribution of the population of normal dust star-forming galaxies, it is crucial to conduct deep and unbiased surveys (continuum and spectroscopic) over large areas with high enough spatial resolution, as the ones we propose to do with AtLAST. Such large, homogeneous survey of DSFGs allows for an angular clustering analysis, a determination of the (photometric) redshift distribution, number count estimates, and many other statistical properties. It will provide a high- *z* counterpart to extensively studied large galaxy samples at low and intermediate redshifts.

Perhaps one of AtLAST’s most important contributions to this field will be to study the role of the environment (voids, filaments, groups, clusters) and the way the evolution of DSFGs varies as a function of this environment. This is particularly complementary in the era of Euclid, LSST, Roman and SKA.

The relatively strong negative K-correction in the sub-mm for high-redshift dusty galaxies has been extensively exploited to efficiently: detect DSFGs (either lensed or un-lensed) out to high redshifts, and study them in various amounts of detail, depending on the sensitivity and angular resolution of the sub-mm observatory used for the study. Currently, the ALMA interferometer has the best of both, it is not, however, able to study large samples due to its small field of view - ALMA is not a survey instrument. To study statistical populations of high- *z* galaxies, and environmental effects on their evolution, a complementary, dedicated type of instrument is required, with a high survey speed, low confusion noise limits, and sufficient sensitivity, which is what AtLAST will deliver.

We aim for a comprehensive multi-band imaging survey, uniquely mapping large parts of the sky to specifically target high- *z* galaxies and map their distribution (noting that this could be done in combination with imaging surveys for other science cases). Using a multi-chroic camera, several of the bands can be observed simultaneously, allowing for accurate spectral slope determinations and photometric redshifts (especially in combination with complementary data available at other wavebands), as the observing conditions will be identical for each of the bands. This will provide a rich catalogue of sources to follow-up with ALMA, JWST, or ELT, but more importantly, yield a large homogeneous sample of galaxies in the early Universe.

Wide-field “blind” spectral line surveys with AtLAST will be crucial for mapping the population of “normal” star-forming galaxies across the cosmic history. These are often too faint in dust emission to be detected in continuum surveys. However, as suggested by recent predictions from simulations (e.g., Lagos *et al.*, 2020), while the cosmic star-forming activity is dominated by sub-mm bright galaxies ( *S*_350GHz_ ≥ 1 mJy), the gas budget of the Universe is dominated by sub-mm faint galaxies (quantitatively: *≈*75% of the gas budget at *z* = 2, and ≥90% at *z ≥* 3). Deep, wide-bandwidth spectroscopic surveys of CO and [CII] emission with AtLAST will be critical for mapping the evolution of cold-gas content across the cosmic history. Especially [CII] is a very valuable tracer, physically. It correlates well with the star formation rate ( De Looze *et al.*, 2014), is a valid tracer of the bulk of the gas mass (see Wolfire *et al.*, 2022 for a recent review, and Zanella *et al.*, 2018 for a high-z empirical study), and it is one of the brightest FIR lines out to high-z, as demonstrated by the detection rate of recent ALMA Large Programs, e.g. ALPINE ( Béthermin *et al.*, 2020; Faisst *et al.*, 2020) and REBELS ( Bouwens *et al.*, 2022; Inami *et al.*, 2022).

In the following we explore two worked examples of surveys that AtLAST will make possible. Often a ’wedding-cake" approach is followed, where several surveys are planned with different angular sizes and sensitivity limits, each forming a layer of an imaginary wedding cake. Our two worked examples form the two extremes: the bottom and top layers of the cake, but we will certainly consider the other layers as well, although these could hit the confusion limit for the longer wavelengths if the survey area is too small. An additional pointed survey of galaxy clusters is discussed in Section 2.4.1. With respect to the continuum survey, we list a likely set of frequency bands with associated sensitivities and beam sizes for AtLAST in the companion high- *z* paper by Di Mascolo *et al.* (2024). These are optimized for a range of AtLAST science goals, including the ones presented in this paper.

**Figure 2.**
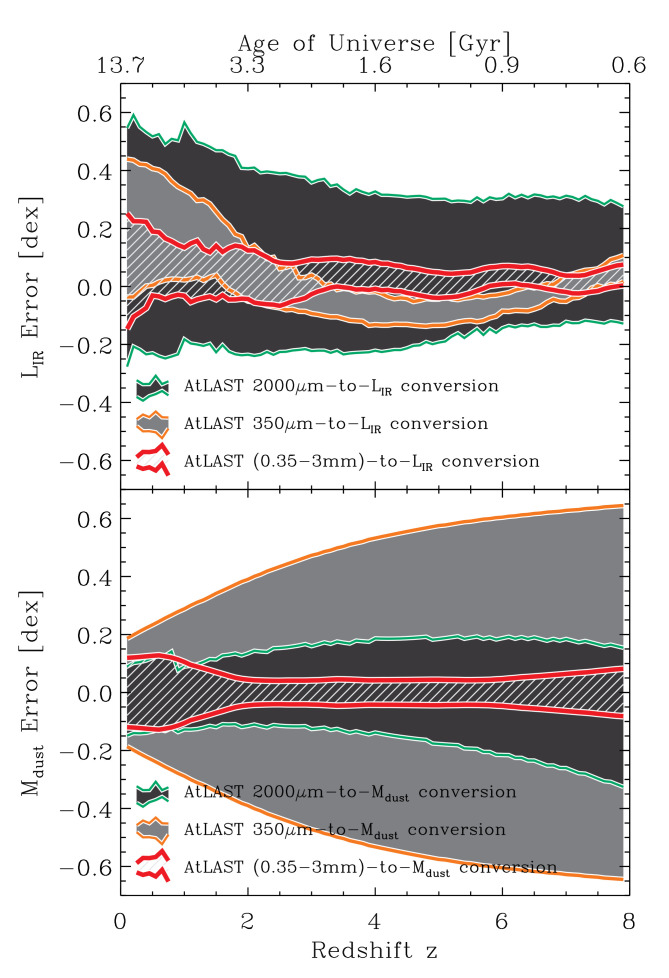
The mean (top panel) and dispersion (bottom panel) of log _10_(L _*IR−BB*_ / L _*IR−True*_) and log _10_(M _*dust−BB*_ / M _*dust−true*_) for mock observations of the SED library of Draine & Li (2007).

***2.1.1. A wide continuum survey.*** To estimate what can actually be achieved with AtLAST, we consider a 1000 deg ^2^ mock galaxy survey as could be obtained in 1000 hours of observing time in the continuum in two or more bands. Such a continuum survey allows one to infer two important physical properties of galaxies: their infrared luminosities L _*IR*_ (and thus SFR _*obscured*_) and dust masses (subsequently M _*ISM*_, assuming a given gas-to-dust ratio). To demonstrate the advantage of a multi-wavelength approach, we estimated the accuracy one would achieve while deriving L _*IR*_ and M _*dust*_ as a function of redshift for different available bands. After exploring almost all possible band combinations, for the purposes of this work we considered the following three cases: (i) galaxies only detected at 2000 *µ*m, (ii) galaxies only detected at 350 *µ*m, and (iii) galaxies detected at (350 or 450) *µ*m and (750 or 850 or 1100) *µ*m and (1300 or 2000 or 3000) *µ*m. To infer these accuracies, we assumed that the diversity of SEDs in the Universe is given by the SED library of Draine & Li (2007), and we fitted mock observations (assuming signal-to-noise ratios of 5) of these templates with a blackbody function (T _*dust*_ in the range 10–60K). In Figure 2 we show the mean and dispersion of the log _10_(L _*IR−BB*_ / L _*IR−True*_) and log _10_(M _*dust−BB*_ / M _*dust−true*_). This shows that 350 *µ*m is a good L _*IR*_ proxy from *z ∼*1-8 (i.e. probing the rest-frame 40–120 *µ*m range of the SED), but a poor proxy for the dust mass. 2000 *µ*m tells the opposite story, i.e., a good dust mass proxy (because of the Rayleigh-Jeans tail) but a poor L _*IR*_ proxy. Finally, one can do very well for both L _*IR*_ and the dust mass if data in more than two bands are available, i.e. the third case we considered. This is quantified in Figure 2: the range of *T _dust_* explored is that of the Draine & Li (2007) SED library, so it does contain SEDs with high temperature. For detection, we used a signal-to-noise limit of 5, and the red lines in 2 show that do better than 0.1 dex (25%) at *z >* 1.

**Figure 3.**
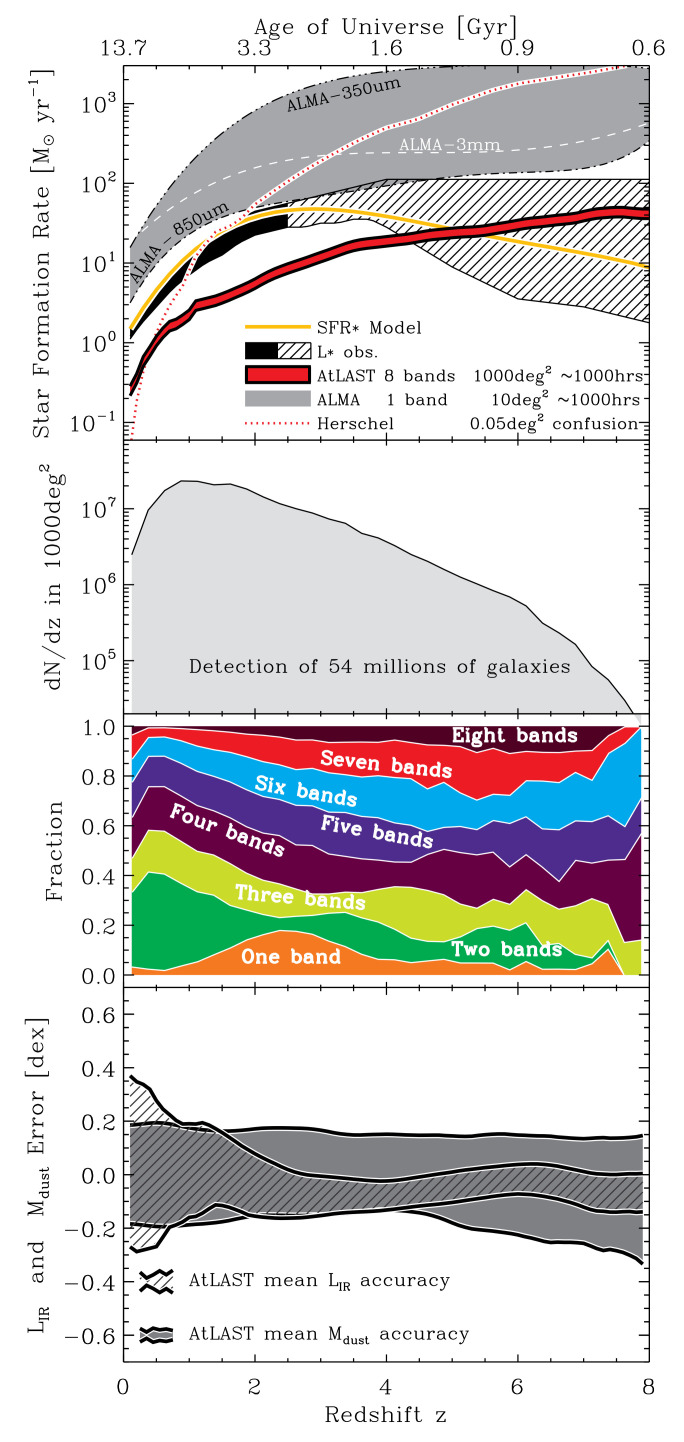
Exploring the parameter space of a 1000 deg ^2^ survey using 1000 hours of AtLAST observing time. Top panel: SFR vs. redshift survey limit. Second panel: *dn/dz*. The third panel shows the fraction of (mock) sources detected on 1, 2, 3, ..., or all 8 bands. The bottom panel shows the expected AtLAST accuracy in inferring *L _FIR_* and *M _dust_* as a function of redshift (better than 25% and 50% at *z >* 2, respectively), demonstrating why multi-band detections are important.

Using the expected mapping speed of AtLAST (fitted with a multi-chroic camera with a million pixels, which is what we expect for our first generation camera), a 1000 deg ^2^ survey (with 1000 hours observing time) results in a 3 *σ* sensitivity limit of 570 *µ*Jy at 350 *µ*m (at this limit, 82% of the Cosmic Infrared Background at 350 *µ*m will be resolved into individual sources) and 324 *µ*Jy at 450 *µ*m. At lower frequencies we hit the confusion limit (e.g. Blain *et al.*, 2002). Note that this implies that going for a much smaller field will only benefit the 350 *µ*m and and 450 *µ*m bands, and thus low-redshift galaxies science, where much work has already been done. With these limits we use the model of Béthermin *et al.* (2017) to explore the parameter space probed by this survey, which is shown in Figure 3.

The first panel of Figure 3 shows the classic star formation rate (SFR) versus redshift survey limit using the models of Béthermin *et al.* (2017) and classical scaling relations. AtLAST will be able to detect sources below SFR _∗_ up to z *∼*5, where SFR _∗_ is the characteristic SFR defined as the value at the ’knee’ of the classical Schechter function. We compare this to what Herschel and ALMA can do: for ALMA we considered a hypothetical 1000 hours survey in one ALMA band only (either 350 *µ*m, 450 *µ*m or 3mm), over 10 deg ^2^ (requiring tens of thousands of pointings at 0.85–1.3mm), assuming that the ALMA sensitivity will be twice as good as it is now (bandwidth increased by a factor 4, taking the ALMA WSU upgrade into account: Carpenter *et al.*, 2023) and no overheads. The shaded region shows the range of star formation rates probed by such a survey. ALMA, with its small field of view, is clearly not efficient. The most optimal survey with ALMA would be at 850um, still barely reaching SFR _∗_ up to *z ∼*4. The second panel of Figure 3 displays *dn/dz* for our 1000 deg ^2^ survey, whereas the third one shows the fraction of sources with detection in only 1 band, only 2 bands, etc., up to all 8 bands. Over the full redshift range, about 70–80% of our galaxies will have detection in at least 3 bands, and therefore can be used to simultaneously solve for L _*IR*_ and dust mass, using photometric redshift estimates from AtLAST itself and elsewhere.

With a facility like AtLAST, we will, for the first time, have the far-infrared SED (L _*IR*_, M _*DUST*_, T _*DUST*_) of all the relevant star-forming galaxies (SFR larger than halve of SFR _∗_) from *z ∼*0 to *z ∼*5–6. This will allow statistically-sound studies on the star forming and inter-stellar matter content of galaxies even while dividing our sample in many redshift, mass, metallicity, environment, and morphology subsamples. Such a survey will prove extremely valuable to complement already planned large optical/near-infrared surveys from which our photometric redshifts will be drawn (combined with the multi-band AtLAST data). The bottom panel of Figure 3 shows the average L _*IR*_ and M _*DUST*_ accuracies of our survey. This quantifies why having detections in several bands for most of our galaxies is important (70–80% of the galaxies will have at least bands, as shown in the third panel). We will have an accuracy better than *∼*0.3 dex for both L _*IR*_ and M _*DUST*_ for over 54 million galaxies, which will be unprecedented. This survey does need good 350/450 *µ*m conditions, so observing should be spread over four years at least, most likely. Interestingly, this continuum survey will contain many Virgo/Coma-like structures up to *z ∼*2, and group/poor clusters up to *z ∼*6. We discuss how to obtain a large, complete cluster sample in Section 2.4.

***2.1.2 A deep “blind” spectroscopic survey*.** The high density of spectral features and large spectroscopic bandwidths of optical spectrographs make the optical regime an excellent wavelength range to determine the redshifts of large samples of galaxies. For example, one of the most ambitious new redshift surveys will be done using *Euclid*, with a target number of 1.5×10 ^8^ galaxy redshifts ( Laureijs *et al.*, 2011). However, optical redshifts provide a biased view, missing most of the dust obscured objects. This is particularly important for the most highly star-forming objects which tend to be obscured by their large reservoirs of interstellar dust. The redshifts of these dusty star-forming galaxies (DSFGs) have been first attempted with optical spectroscopy (e.g. Chapman *et al.*, 2005), but it has since become clear that direct mm spectroscopy is a much more exact and efficient method (e.g. Chen *et al.*, 2022; Cox *et al.*, 2023; Reuter *et al.*, 2020; Weiss *et al.*, 2007).

Therefore, in addition to a very wide continuum survey, we also estimate what a deep line survey with AtLAST can achieve. We consider a factor of three increase in time (3000 hours), which is realistic as a line survey uses mostly the low-frequency part of the spectrum: the 350/450 *µ*m bands do not provide many lines but for CII at *z ∼*1.5–2.0. The exercise here is to see what a 3000 hours spectrocopic survey with AtLAST delivers.

Again using the expected mapping speed of AtLAST, for a deep 1 deg ^2^ line survey (with 3000 hours observing time) in a 400km/s channel (R=750), we can estimate the sensitivity limits for the various typical bands. Not listing all available bands, we find (assuming no confusion) 3 *σ* sensitivity limits ranging from 2330 *µ*Jy at 350 *µ*m, 210 *µ*Jy at 850 *µ*m, to 27 *µ*Jy at 3 mm. With these limits we employ the model of Béthermin *et al.* (2017) to model the galaxy population and predict their CO and fine structure line (FSL) peak flux densities, assuming a line width of *∼*400 km/s (i.e., matching our channel width). We do this for the CO lines from J _*up*_ =1 to J _*up*_ =7, assuming L’ _*CO*(1−0)_/L _*IR*_ = 40 and a sub-mm galaxy CO ladder as in Tab. 2 of Carilli & Walter (2013). We take L _*CII*_/L _*IR*_ *∼* 3 × 10 ^−3^ (not in the deficit part as our survey will be dominated by SFR _∗_ galaxies). Other FSLs are CI _610_, CI _370_, NII _205_, OI _146_, NII _122_, OIII _88_, OI _63_ and OIII _51_, for which we use an FSL _*line*_/L _*IR*_ ratio as found in the literature Graciá-Carpio *et al.* (2011), Bothwell *et al.* (2016), Zhao *et al.* (2016), Carilli & Walter (2013), Schimek *et al.* (2024).

**Figure 4.**
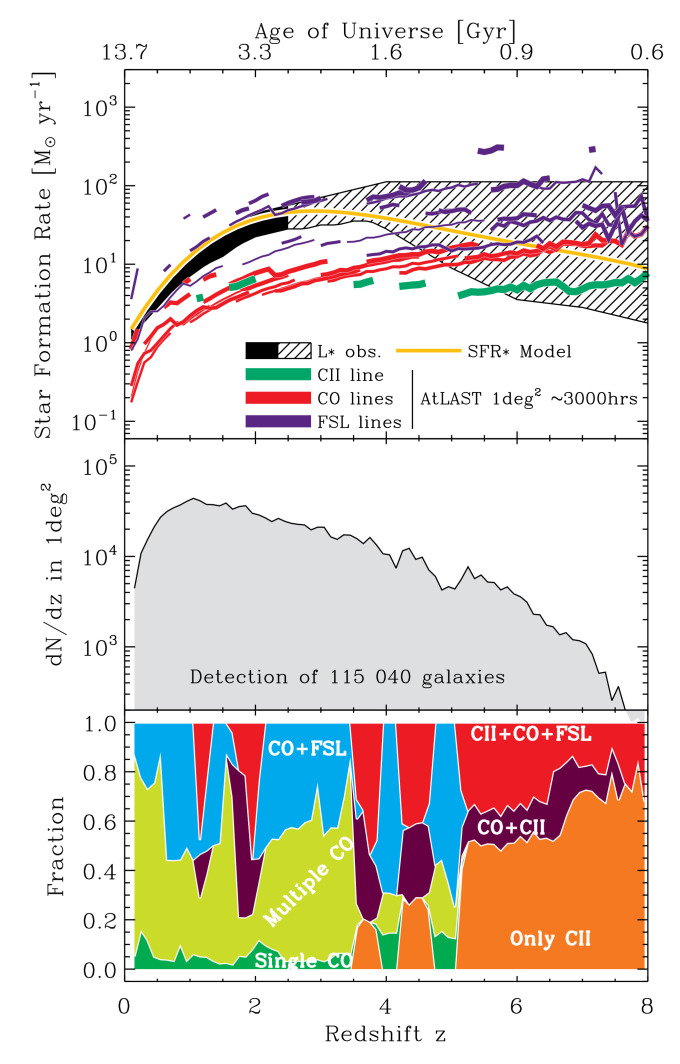
Exploring the parameter space of a 1 deg ^2^ deep line survey using 3000 hours of AtLAST observing time. The top panel shows, for each line considered, the minimum star formation rate (SFR) a galaxy must have for be detected at a given redshift (see main text for more details). The second panel displays *dn/dz*. The third panel shows the fraction of (mock) sources with only CII detection, only a single CO detection, only multiple CO detection (no CII, no FSL).

For this survey setup we again plot the parameter space probed by this line survey, now in Figure 4. The top panel shows, for any given line considered, the minimum SFR a galaxy must have to be detected at a given redshift (for CO, the thin line is for J=1-0, and the thickest line for J=7–6; for FSL, the thin line is for CI610, and the thickest line for OIII51; i.e. plot line thickness increases with increasing energy). Such a survey will basically provide multiple line detection for galaxies below SFR _∗_ up to *z ∼*7. In particular, SFR _∗_ galaxies at 4 *< z <* 7 should all have one CO detection and one [CII] detection, reminding ourselves that multiple line detection is crucial for redshift determination. Of course, [CII] at high redshift detects sources well below SFR _∗_. One can argue that using energetic considerations, such single line detections could be used on their own to constrain the redshift of these sources. Note that ALMA will be a factor *∼* 50 less sensitive for a 3000 hrs / 1 deg ^2^ survey. The second panel of Figure 4 shows *dn/dz* for our 1 deg ^2^ mock line survey. Finally, the bottom panel of Figure 4 shows the fraction of sources with only [CII] detection, only a single CO detection, and only multiple CO detection (no [CII], no FSL). About 90% and 50% of our galaxies at *z <* 5 and *z >* 5, respectively, will have multiple line detections, which is excellent.

All this means that an AtLAST 1 deg ^2^/3000 hrs line survey (a 400 km/s channel, i.e. R=750) will basically give us multiple line detection for SFR _∗_ galaxies up to *z ∼*7. Combining this with multiple band continuum detections will allow us to obtain a wealth of information for each of these SFR _∗_ *z <* 7 galaxies: the redshift, gas content, cooling budget, star formation rate, dust mass, and dust temperature. A survey going much wider than 1 deg ^2^ in the same amount of time will loose many of the CO lines, but will still detect [CII].

### 2.2 Constraining cosmological parameters via BAO and clustering

One of the most fundamental issues in modern cosmology is the significantly different values of the Hubble constant measured from the CMB (the early universe) and those based on late time observations, colloquially known as ’Hubble tension’ (see Di Valentino *et al.*, 2021 for a review). Improving and expanding the state-of-the-art measurement methods is one way to tackle the Hubble tension. Clustering of galaxies measured in large samples with spectroscopic redshifts provides a powerful means in this regard, and has been shown to be one of the standard references since the first significant measurements of the redshift space distortion (RSD; Peacock *et al.*, 2001) and the Baryon Acoustic Oscillation (BAO, sound waves from the embryonic Universe; Cole *et al.*, 2005). This effective approach has been realized and the cosmological parameters, especially the growth rate, f *σ*_8_, and the Hubble constant, have been measured to percent level precision up to *z* ≲ 1 ( Bautista *et al.*, 2021; DES Collaboration *et al.*, 2024), and the new surveys that will be carried out by Dark Energy Spectroscopic Instrument and the Subaru Prime Focus Spectrograph will push that limit to *z ∼* 2.4 ( DESI Collaboration *et al.*, 2016; Takada *et al.*, 2014).

Similar measurements beyond *z ∼* 2.4 may start to become challenging for optical and near infrared observations, since most of the strong lines move toward longer wavelengths that are hard to access from the ground, and as galaxies become fainter at higher redshifts and so do their lines. Measurements in the FIR, on the other hand, become more easily accessible thanks to the shifting of the bright [CII] and CO lines to the more transparent atmospheric windows. Indeed, detecting these far-infrared fine-structure lines from galaxies at *z >* 2 has become a regular practice for extragalactic studies in the early cosmic time (e.g., Béthermin *et al.*, 2020; Bouwens *et al.*, 2022)

A high-redshift galaxy spectroscopic survey with AtLAST would combine the successful strategy from the optical/infrared surveys that measured galaxy clustering in the last few decades (eg. Amvrosiadis *et al.*, 2019; Cochrane *et al.*, 2018; Johnston *et al.*, 2021; Vakili *et al.*, 2023; van Kampen *et al.*, 2023; Wilkinson *et al.*, 2017, and many references therein) with the new possibility of measuring strong emission lines from the FIR regime for large samples of galaxies at *z* ≳ 3.

To test the feasibility and help design such a survey, as a first step, we have conducted a simple forecast study using dark matter only cosmological simulation at *z* = 3. We use *N* -body simulation runs that are part of the D ARK Q UEST project ( Nishimichi *et al.*, 2019). The simulation box has 2 Gpc/h on a side with the Planck cosmology as the fiducial cosmological model. Halos are identified using the ROCKSTAR algorithm ( Behroozi *et al.*, 2013). The halo mass is defined by a sphere with a radius *R*_200m_ within which the enclosed average density is 200 times the mean matter density, as *M _h_ ≡ M*_200m_. Motivated by recent clustering measurements of dusty star-forming galaxies at *z >* 1 ( Lim *et al.*, 2020; Stach *et al.*, 2021), we apply a selection of halos with masses of 10 ^12.5^ − 10 ^13.5^ solar masses, which yields about 800k halos for the analyses.

The two-point correlation functions are measured using the selected simulated halos, and covariance matrix is estimated with the standard bootstrap method ( Norberg *et al.*, 2009). The models considered for the fitting of the correlation functions are the fiducial Λ-CDM, Baryonic Accustic Occasilations (BAO; Eisenstein *et al.*, 2005), linear redshift space distortion (RSD; Kaiser, 1987), and the Alcock-Paczynski effect ( Alcock & Paczynski, 1979). In this model, we have four free parameters in total, bias ( *bσ*_8_), growth rate ( *f σ*_8_), Hubble parameter ( *H*( *z*)) and angular diameter distance ( *D _A_*). To break the degeneracy between bias and the growth rate, we perform fittings of the monopole and the quadrapole moments of the measured correlation functions, over a pair separation range of 25–140 Mpc/ *h* and 40–140 Mpc/ *h*, respectively.

**Figure 5.**
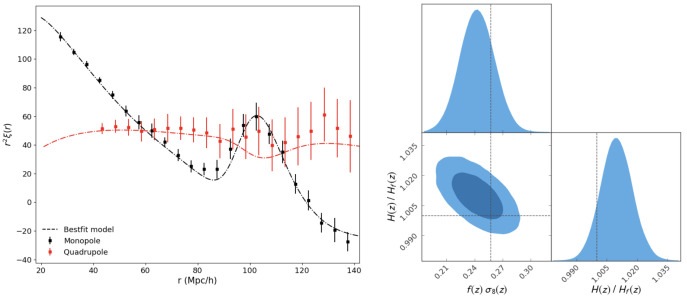
*Left:* Data points with errors are monopole and quadrupole moments of the correlation functions measured using dark matter halos in a simulated dark matter only box, and the curves are the best-fit models including four parameters, *f σ*_8_, *bσ*_8_, *H*( *z*) and *D _A_*. Both BAO and RSD are considered in these fittings. Data points are shown within the separation ranges on which the fittings are performed. *Right*: The results of the MCMC analyses with one and two sigma confidence regions colour coded by different darkness of blue. Here *bσ*_8_ and *D _A_* are marginalized over.

The results are plotted in Figure 5, where the left panel shows the results of the fittings of the correlation functions and the right panel shows the MCMC results of the cosmological parameters, including the Hubble parameter and the growth rate. The other two parameters, bias and angular diameter distance, are marginalized over. In summary, the Hubble parameter can be measured at 0.7% precision and the growth rate at 7.3%.

The above analyses demonstrate the potential constraining power of a baseline design of a AtLAST spectroscopic survey; that is, a spectroscopic survey of hundreds of thousands of sources with a footprint of about 1000 square degrees, which can be achieved with a survey of a few thousand hours when employing the bright [CII] line, as shown in the previous sections.

### 2.3 Line-intensity mapping (tomography)

A novel method for studying the large-scale structure of the Universe is the line-intensity mapping (LIM) of the [CII] and CO emission lines. Specifically, line-intensity mapping measures 2- and 3-dimensional power spectra from spectral cubes, providing a statistical view of the large-scale structure across cosmic time. LIM experiments at sub-mm wavelengths bridge the optical surveys at *z ≤* 1 and the upcoming radio surveys of the 21-cm HI emission line in the Epoch of Reionisation ( *z ≥* 6). Namely, the optical surveys become inefficient at higher redshifts as the bright optical lines move into infrared wavelengths and the dust obscuration increases; conversely, the 21-cm line signal peters out at *z ≤* 6 as the neutral hydrogen in the intergalactic medium becomes fully ionised. The [CII] and CO lines remain bright across this redshift range and do not suffer from dust obscuration, making them ideal tracers of the large-scale structure.

**Figure 6.**
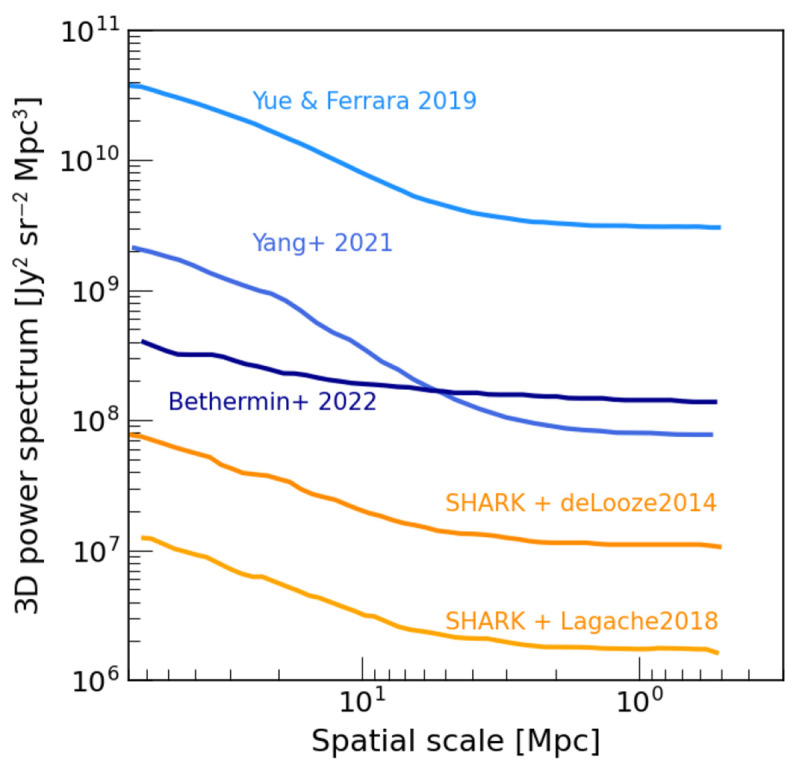
Line-intensity mapping: predicted 3D power spectrum of the [CII] emission at redshift *z* = 3. Individual curves correspond to different analytical ( Yang *et al.*, 2021; Yue & Ferrara, 2019) and N-body + semi-analytical models ( Béthermin *et al.*, 2022; Lagos *et al.*, 2020). We also show two realisations of the SHARK semi-analytical model coupled with two different prescriptions for [CII] emission ( Lagache *et al.*, 2018). The predictions vary by over 3 dex; the large FoV and sensitivity of AtLAST will be critical for measuring LIM signal across cosmic time. Adapted from Béthermin *et al.* (2022), J. Hilhorst (MSc thesis).

***2.3.1 Predictions for LIM signal.*** The predictions for LIM mapping signal (and the associated foregrounds) have been explored theoretically using different approaches: from simple analytical models (e.g., Yue & Ferrara, 2019) to dark-matter only simulations combined with semi-analytical models (e.g., Béthermin *et al.*, 2022) and cosmological-volume hydrodynamical simulations (e.g., Karoumpis *et al.*, 2022). The predictions for the resulting 2D and 3D power spectra vary by *several orders of magnitude* ( Figure 6), chiefly due to differences in the assumptions on galaxy evolution and predictions of emission line intensity.

***2.3.2 Current observations: lack of constraints.*** Several teams have conducted early LIM experiments on 10-metre class telescopes. CONCERTO, a scanning Martin-Puplett interferometer with MKID detectors covering 125 – 310 GHz frequency range, was installed on the APEX telescope in 2021 ( CONCERTO Collaboration *et al.*, 2020; Monfardini *et al.*, 2022), mapping *≈*1.4 deg ^2^. TIME is 16-pixel grating spectrometer observing the 200–300 GHz band, mounted on the 12-m ARO telescope ( Crites *et al.*, 2014; Li *et al.*, 2018). At lower redshifts, COMAP ( Cleary *et al.*, 2022; Lamb *et al.*, 2022) is a 19-pixel heterodyne spectrometer mapping the CO(1–0)/(2–emission lines in the 26–34 GHz band. These experiments are currently limited by the small collecting areas, low pixel count, and small survey areas (few deg ^2^); rather than detecting the LIM signal at high redshift, they will provide upper bounds, potentially ruling out some of the more “extreme” models. AtLAST will supersede these facilities by providing a much larger collecting area, large focal plane, and superior site and dish quality.

One of the key challenges in measuring the 2/3D power spectra of, e.g., [CII] emission, is the need to remove “interlopers”: either CO and [CI] lines from lower-redshift galaxies, or [OIII] emitters at higher redshifts. This can be achieved by several techniques, such as masking (known) foreground galaxies (e.g., Béthermin *et al.*, 2022) or using the periodicity of CO emission lines to separate the CO and [CII] power spectra (e.g., Yue & Ferrara 2019).

**Figure 7.**
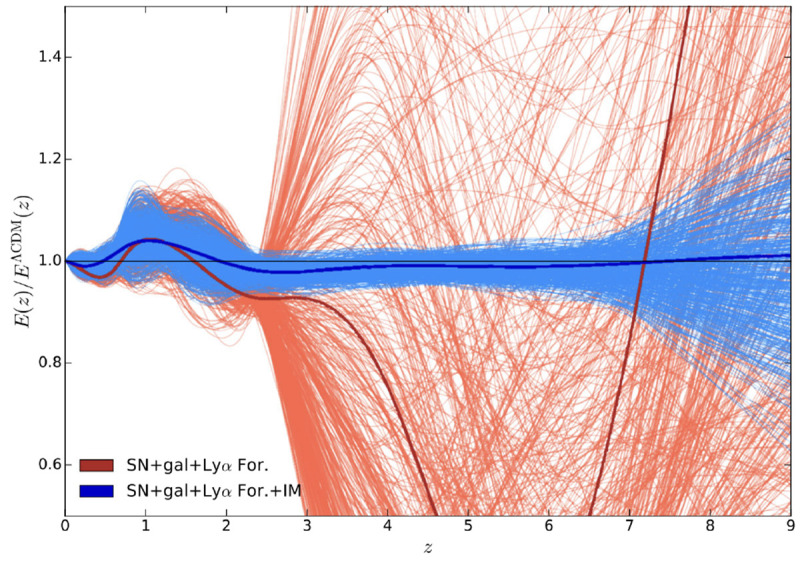
Model-independent constraints on the shape of the cosmic expansion history, *E*( *z*) = *H*( *z*) */H*_0_ (normalised to *Λ*CDM), with (blue lines) and without (red lines) CO(1-0) Line-Intensity Mapping over a 1000 deg ^2^ patch. For details, see Bernal *et al.* (2019) and Silva *et al.* (2021), from which this figure was taken. It emphasizes the importance of LIM in constraining the cosmic expansion history, especially at *z >* 3.

The power of CO LIM to constrain the cosmic expansion history *H*( *z*), i.e the Hubble expansion rate as a function of redshift, is illustrated in Figure 7, especially for *z >* 3. This figure, taken from Silva *et al.* (2021), and based on the work of Bernal *et al.* (2019), compares using only Supernovae (SN), galaxy surveys and the Lyman- *α* forest (red lines) to a combination of these with a LIM experiment measuring CO(1-0) over a 1000 deg ^2^ area. These calculations were not specifically performed for AtLAST, but do illustrate well the how much LIM with AtLAST can contribute to constraining the cosmic expansion history.

### 2.4 Surveying cluster galaxies in the distant Universe

**Figure 8.**
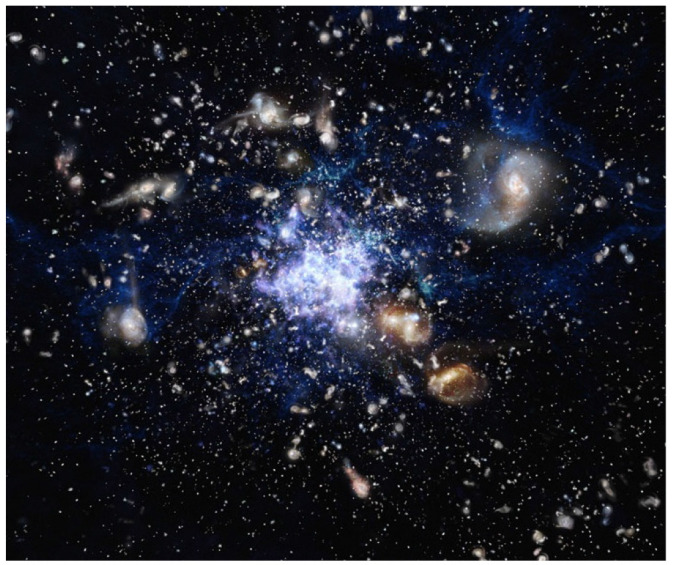
This artist’s impression depicts the formation of a galaxy cluster in the early Universe. The galaxies are vigorously forming new stars and interacting with each other. They are observed as Far-Infrared/Sub-mm Galaxies or Dusty Star-Forming Galaxies. Credit: ESO/M. Kornmesser. Courtesy from ESO Press Release October 2014.

Galaxy clusters are the first large structures to form and eventually evolve into the largest virialised objects in the Universe. They should therefore be seen as the earliest fingerprint of galaxy formation and evolution (e.g. the review of Kravtsov & Borgani, 2012). Clusters grow hierarchically through the merging and accretion of smaller units of galaxy halos, which are dominated by (very) young galaxies displaying intense bursts of star-formation — the dusty star-forming galaxy population (DSFGs; see for a review Casey *et al.*, 2014). These are rich in molecular gas but also heavily obscured by dust, which makes them prime targets for far-infrared/submm facilities ( Alberts & Noble, 2022). These early cluster galaxies are most probably the progenitors of elliptical galaxies (eg. Ivison *et al.*, 2013; Lutz *et al.*, 2001) which end up dominating local galaxy clusters. Figure 8 (based on work by ( Dannerbauer *et al.*, 2014) on a *z* = 2.2 proto-cluster) shows how violent galaxy clusters could be in the distant Universe. In this section we motivate the need of a systematic study of the early galaxy population in proto-clusters in order to make big leaps forward in this emerging research field, which we argue is best done with a large single dish sub-mm telescope at a high site. Please note that we focus here on the cluster galaxy population: a companion AtLAST case study by Di Mascolo *et al.* (2024) focuses on probing the ICM using the full SZ spectrum.

***2.4.1 A systematic mapping survey of distant cluster galaxies.*** Presently, samples of galaxies in proto-clusters are small and heterogenous, cannot sample the full extent of the cluster infall regions, and take a lot of time to complete using current facilities. For this reason there are still relatively few cold ISM measurements of cluster galaxies. A future systematic (sub-)mm survey of high-redshift (proto-)galaxy clusters will resolve this situation, and allow us to answer the following scientific questions in detail: 1) How do galaxies and clusters co-evolve at early times? 2) How does environment (especially in over-dense regions) affect star formation, enrichment, outflows and feedback processes? and 3) What is the time evolution of each of these processes? When and where do they peak?

Currently the number of known spectroscopically confirmed (proto-)clusters beyond *z* = 2 is still relatively low (see for a review Overzier, 2016), where the targets are often sparsely sampled. The heterogeneous datasets collected so far suffer from strong selection biases and projection effects (e.g. Chen *et al.*, 2023), which prevent obtaining a complete picture of the build-up of the cluster galaxy population over cosmic time. To remedy this, a large and statistical sample is required. However, each of these clusters and their infall region cover a linear extension of up to 30 Mpc ( Casey, 2016; Lovell *et al.*, 2018; Muldrew *et al.*, 2015), which corresponds to about 30 *′* at *z ∼* 1 − 7. Therefore, to study and understand the epoch of cluster formation, one really needs to cover areas of up to one square degree, something current sub-mm facilities cannot practically do with adequate sensitivity and survey speed.

For example, ALMA allows for a survey speed of at most a few square arcminutes per hour for the brightest CO lines ( Popping *et al.*, 2016) to yield detections for a sufficient number of cluster galaxies with L> L _∗_ at *z ≈* 1. The survey speed for a given line depends on the sensitivity to that line and the primary beam at its frequency. This determines the number of pointings required for a given desired map size: obtaining an area of a square degree with ALMA would take at least 1000 hours for the line with the highest survey speed, CO(5-4). This line is hard to interpret physically, while CO(3-2) would take around 6000 hours with ALMA, and CO(2-1) would even need three times that ( Popping *et al.*, 2016). This renders one degree surveys for the lower transition lines prohibitively expensive with ALMA. Another property of ALMA is the relatively narrow bandwidth (just below 8 GHz) which makes spectral scans slow as well.

The past decade has seen a rise in several hundred detection’s of the cold molecular gas supply that fuel the star formation in the distant Universe, albeit focusing mostly on isolated field galaxies ( Carilli & Walter, 2013; Tacconi *et al.*, 2018). However, the number of published cold gas measurements of galaxies located in galaxy clusters at *z >* 1 is fairly low (of order a hundred). Even though ALMA and ATCA allowed this number to increase significantly ( Alberts & Noble, 2022; Coogan *et al.*, 2018; Cramer *et al.*, 2023; Dannerbauer *et al.*, 2017; Hayashi *et al.*, 2017; Hayashi *et al.*, 2018; Jin *et al.*, 2021; Noble *et al.*, 2017; Noble *et al.*, 2019; Rudnick *et al.*, 2017; Stach *et al.*, 2017; Tadaki *et al.*, 2019; Williams *et al.*, 2022), it remains low nonetheless. In order to resolve these problems described above, we aim to produce a high-redshift counterpart to local large cluster galaxy surveys (eg. Abell *et al.*, 1989). This will help us understand the contribution of protoclusters to the obscured cosmic star formation rate density evolution ( Chiang *et al.*, 2017).

**Figure 9.**
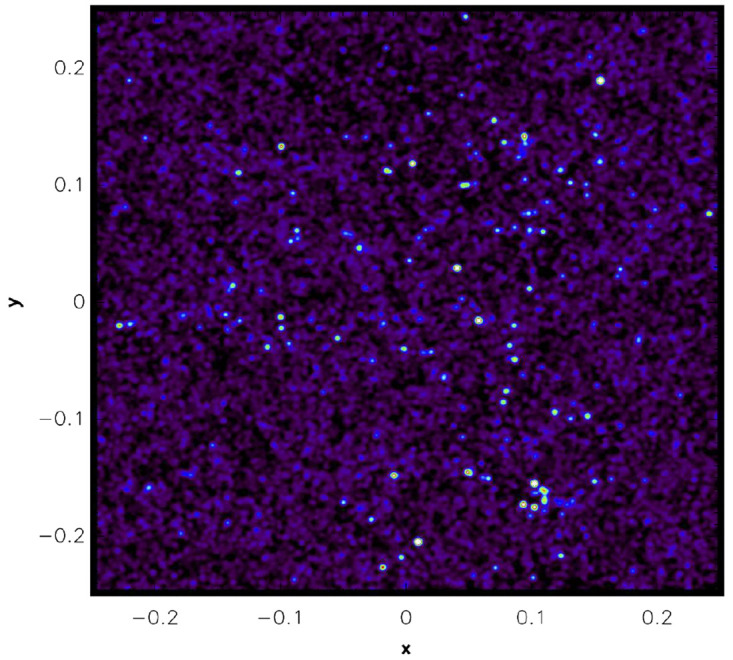
Example mock image at 2.4 mm of the CO(3-2) flux of galaxies in and around a simulated *z* = 1.74 proto-cluster, including homogeneous background noise and field galaxies along the line-of-sight. This 0.5 × 0.5 degree image constitutes a single pointing with a single-dish 50 m sub-mm telescope.

***2.4.2 The way forward.*** In order to make a big leap forward in understanding the evolution and formation of the largest structures and galaxies, a sub-mm observatory optimized for surveys is needed, i.e. a highly multiplexed instrument on a telescope with a single dish of at least 50 m. To visualize what such a telescope can achieve, a mock CO(3-2) image of a simulated proto-cluster at *z* = 1.74 is shown in Figure 9 (homogeneous noise is added), observed in a single pointing at 2.4 mm where the angular resolution would be of order of 12 arcsec for such a telescope, and up to 6× better at higher frequencies. This mock image is derived from a light-cone constructed out of a semi-analytical galaxy formation model ( van Kampen *et al.*, 2005) in which a cluster simulation (using the same model set-up) was inserted at *z* = 1.74 (a few hundred cluster galaxies were added in this way, of which around 50 are sufficiently bright to be detectable at the depth of this particular mock image).

We will target galaxies in already confirmed galaxy clusters (with known spectroscopic redshifts) as well as candidate clusters. In addition, we expect to discover new proto-clusters, especially dust obscured ones with large molecular gas reservoirs contained in their individual members, in the large surveys planned for AtLAST. At the time of conducting this survey with a large single dish telescope, a sample of several to a few ten thousand (proto-)galaxy clusters from *z* = 1 − 10 will exist coming from future surveys and missions such as LSST and Euclid, the latter one with a dedicated study to discover galaxy clusters at high redshift ( Laureijs *et al.*, 2011). Presently we already have a few thousand known candidate (proto-)galaxy clusters from Planck ( Planck Collaboration *et al.*, 2015; Planck Collaboration *et al.*, 2016) and Hyper Suprime-Cam ( Higuchi *et al.*, 2019; Toshikawa *et al.*, 2018).

## 3 Technical justification

To meet the observational requirements outlined here, we perceive the most salient instrumentation requirements to be the ability to achieve high mapping speeds over large areas, in multiple bands for continuum surveys, and with very wide bandwidths for emission line surveys. The current state of the art Transition Edge Sensor (TES) bolometers or Kinetic Inductance Detectors (KIDs) present the likeliest technological path to achieve this, as both of these are of high technical readiness level, have demonstrated background-limited performance in the mm/submm, and can be read out in large numbers (tens-hundreds of thousands as noted in Klaassen *et al.*, 2020) through frequency multiplexing, allowing the construction of large imaging arrays and integral field units. In the following subsections we discuss the technical requirements for the science cases covered by this white paper.

### 3.1 AtLAST as a sub-mm redshift machine

Because DSFGs are dust-obscured, their redshifts are best obtained in the mm/sub-mm wavebands instead of the classical optical or infrared parts of the spectrum. As shown by e.g. Bakx & Dannerbauer (2022), a key requirement for such direct mm spectroscopy is a very wide frequency coverage, which is difficult to obtain with heterodyne receivers. Several dedicated broad-bandwidth (but low spectral resolution) instruments were specifically designed for redshift searches on single-dish (sub-)mm telescopes, such as Z-Spec ( Naylor *et al.*, 2003), Zspectrometer ( Harris *et al.*, 2007), ZEUS ( Ferkinhoff *et al.*, 2010), the Redshift Search Receiver ( Erickson *et al.*, 2007), or DESHIMA ( Endo *et al.*, 2019; Taniguchi *et al.*, 2022). These instruments have demonstrated the technical feasibility of innovative technology, but still resulted in only a few dozen new redshifts due to the limited sensitivity of the telescopes they were mounted on. The ALMA Wide-band Sensitivity Upgrade (WSU; Carpenter *et al.*, 2023) will improve ALMA’s capabilities for redshift determinations, especially in Band 2 ( Mroczkowski *et al.*, 2019). However, due to ALMA’s very limited primary beam, redshift searches are done mostly on individual, known targets, or within very small fields.

AtLAST promises to make a leap forward thanks to its unique combination of sensitivity, broad spectral bandwidth, wide field of view and multiplex spectroscopic capabilites. Based on the prototype instruments described above, the MKID-based IFUs will allow to cover frequency ranges of hundreds of GHz. This very broad bandwidth will be possible as the requirements on spectral resolution are rather low: *∼*0.5 GHz will be sufficient to avoid line smearing as the targets will be galaxies with line widths of several hundreds to >1000 km s ^−1^. Rather than covering a single object line the prototype instruments mentioned above, the AtLAST IFUs will eventually cover the full focal plane of 2° diameter.

The very broad spectral bandwidth is crucial for redshift determinations: for example instrument covering the atmospheric windows from 125 to 500 GHz would allow to use the brighter but more widely spaced FSLs rather than the fainter CO lines for redshift determinations (see Figure 6 of the companion case study on CGM science by Lee *et al.*, 2024). Furthermore, FSL would mostly circumvent the potential redshift degeneracies that follow the linear spacing of CO lines. An additional advantage of FSL over the CO ladder is that they cover a wider range of physical conditions in the gas clouds, covering not only the PDR’s but also HII regions (see also Lee *et al.*, 2024); this will allow to obtain a more complete census of the sources in an unbiased wide-field redshift survey. Finally, very broadband IFU studies will not only cover the emission lines, but will also allow to determine the slope of the continuum emission which is brighter at shorter wavelengths, providing an additional constraint on the redshifts.

There exists the possibility for redshift degeneracies in a redshift survey targeting CO lines ( Bakx & Dannerbauer, 2022). To assess this, a tool is available ( https://github.com/tjlcbakx/redshift-search-graphs) to graph the ability of an instrument to derive the redshift of a galaxy in each of the redshift bins using solely the observational bandwidth of a receiver. It can be used to determine the fraction of sources in a redshift regime that will have no lines, one line, or multiple robust lines. Doing this exercise for the 2-3 mm windows shows that that a wide band IFU covering these windows would be highly efficient at *z >* 2.

For the line intensity mapping method we need a broad spectral coverage to map the [CII] power spectrum, whereas we require a spectral resolution Δ *v* = 3000 *km/s* ( *R* = 100) to measure the 3D power spectrum. This can be achieved either with a dedicated low-resolution spectrometer, or by binning higher-resolution spectra.

### 3.2 Surveying proto-cluster galaxies

To significantly increase the number of spectroscopically confirmed (proto-)clusters galaxies requires a fast survey sub-mm telescope of at least 50-m. Such a size guarantees the unambiguous identification of cluster galaxies due to the relatively high angular resolution. To get spectroscopic redshifts of several hundred to thousand member galaxies per cluster, a multiplex instrument with up to several thousand elements per field of view is indispensable. One option would be a heterodyne instrument with a wide field of view and extremely large spectral bandwidth, however the costs would be exorbitant, so this will not be feasible. Thus, we opt for for the MKID bolometer technology which should provide integral field unit spectroscopic capabilities.

To guarantee spectroscopic redshifts from *z* = 1−10 and a complete study of the most prominent lines emitted from the cold ISM such as multiple CO, the two [CI], the [CII], H _2_O and HCN lines, the spectrometer should have an unprecedented bandwidth from 70 to 700 GHz. E.g., the brightest expected line emitted in the far-infrared is [CII] at 158 *µ*m. An instrument with spectral coverage from 180 to 345 GHz could thus follow the early stages of cluster evolution from *z* = 4.5 − 10, whereas extending to higher frequencies ( *∼*700 GHz) would even allow us to map the peak of the star-formation and black hole activity of the Universe at *z* = 2 ( Madau & Dickinson, 2014) with the same line. Furthermore, such a set-up guarantees the detection of several CO lines and the so-called CO SLED (spectral line energy distribution, e.g. Dannerbauer *et al.*, 2009; Daddi *et al.*, 2015) can be established. This enables us to securely determine physical properties such as the gas density, excitation temperature and even molecular gas mass. In addition, with both [CI] lines the total cold gas mass can be measured as well ( Papadopoulos *et al.*, 2004; Tomassetti *et al.*, 2014). Getting a complete picture of the cold ISM supported by a large sample size is indispensable to study in a statistical way if environment plays a role by measuring parameters such as star-formation efficiency, molecular gas fraction and excitation.

We need a fast enough survey telescope that not only allows the study of confirmed galaxy clusters (with known spectroscopic redshifts) and candidate clusters but in addition will yield an unbiased survey (negelecting the impact of cosmic variance) of a significant area of the sky (to beat cosmic variance) within a reasonable time span. Therefore, to conduct the survey and achieve the science goals following technical requirements should be fulfilled:

- a bolometer based on millimeter KID technology with many thousands of elements per field of view, which has spectroscopic properties similar to multi-object spectroscopy in the optical and near-infrared

- a large bandwidth from 70 to 700 GHz to obtain spectroscopic redshifts,

- a field-of-view of *∼*1 square degree to cover the typical size of (proto-)galaxy clusters,

- a spectral resolution of 500 − 1000 km/s to detect cluster galaxies and determine their redshifts (preferably from two or more lines),

-a survey speed of at least 15 arcmin ^2^ per minute to obtain a statistically significant sample of several thousand galaxy clusters.

## 4 Summary and conclusions

In this paper we outline several high-z science cases for AtLAST, a future 50-m submillimeter telescope in the Atacama dessert, focusing on the overall high-z galaxy population as well on galaxies in proto-clusters. Two companion AtLAST case studies focus on emission line probes of the CGM of galaxies ( Lee *et al.*, 2024) and on probing the ICM using the full SZ spectrum ( Di Mascolo *et al.*, 2024).

AtLAST will have high angular resolution, a large collecting area and large focal plane, and therefore a high survey speed (see e.g. Klaassen *et al.*, 2020; Mroczkowski *et al.*, 2023; Mroczkowski *et al.*, 2024; Ramasawmy *et al.*, 2022). This means AtLAST can cover large areas of the sky for high- *z* galaxy surveys. A single pointing will be of order 200 time larger than the LMT, for example. Also, the photometric confusion noise at 350 *µ*m will be four orders of magnitude lower than that of existing 6-m sub-mm telescopes. These mayor improvements on existing facilities, combined with an excellent instrument suite, allows for a large leap forward in the active research field of early galaxy formation and evolution, and notably the study of Dusty Star-Forming Galaxies (DSFGs).

One or more large, homogeneous surveys (continuum or spectral line) of DSFGs will yield classical statistical properties such as the auto-correlation function, the (photometric) redshift distribution, number counts, and so forth. It will also yield a high- *z* counterpart to existing large galaxy samples at low and intermediate redshifts, and make mayor contributions to the understanding of how the evolution of DSFGs varies as a function the environment (voids, filaments, groups, clusters). Additionally, such surveys provide a rich catalogue of sources to follow-up with ALMA, JWST, and ELT.

Using a large multi-chroic camera allows for a comprehensive multi-band imaging survey, uniquely mapping large parts of the sky to specifically target high- *z* galaxies and map their distribution, where several of the bands can be observed simultaneously, allowing for accurate spectral slope determinations and photometric redshifts (especially in combination with complementary data available at other wavebands), for example. A worked example for a 1000 deg ^2^ continuum survey in 1000 hours was presented, which for AtLAST will have a 3 *σ* sensitivity limit of 570 *µ*Jy at 350 *µ*m (at this limit, 82% of the Cosmic Infrared Background at 350 *µ*m will be resolved into individual sources) and 324 *µ*Jy at 450 *µ*m. We showed that the multi-wavelength approach significantly increases the accuracy one would achieve while deriving L _*IR*_ and M _*dust*_ as a function of redshift for different available bands, especially if data for three or more bands is available.

In addition to a very wide continuum survey, we also considered a deep line survey with AtLAST using mostly the low-frequency part of the spectrum, which includes [CII] all the way to *z ∼*8. For a deep 1 deg ^2^ line survey in 3000 hours, in a 400km/s channel (R=750), we can go down to 3 *σ* sensitivity limits of 2330 *µ*Jy at 350 *µ*m and 27 *µ*Jy at 3 mm. The model of Béthermin *et al.* (2017) was used predict peak flux densities for CO and various fine structure lines, and found that about 90% and 50% of our galaxies at *z <* 5 and *z >* 5, respectively, will have multiple line detections, which is crucial for redshift determination. Combining this with multiple band continuum detections will allow us to obtain a wealth of information for each of these galaxies besides the redshift: gas content, cooling budget, star formation rate, dust mass, and dust temperature. Therefore, such deep, wide-bandwidth spectral line surveys with AtLAST will be crucial for mapping the population of “normal” star-forming galaxies and their gas content across the cosmic history. Also note that especially the [CII] line will be very promising for this purpose as it is one the brightest FIR lines out to high- *z*.

Besides studying the overall galaxy population at high redshifts, we can also use wide and deep surveys of these galaxies to extract cosmological parameters, especially the growth rate, f *σ*_8_, and the Hubble constant, by measuring galaxy-galaxy clustering (including their monopole and quadrupole moments) and fit cosmological models to these data. At *z ∼* 2.5 this becomes difficult in the traditional optical/infrared bands, but high-redshift galaxy spectroscopic survey with AtLAST would move the successful strategy of the optical/infrared surveys to the mm/sub-mm part of the spectrum and combine this with the new possibility of measuring strong emission lines from the FIR regime at *z* ≳ 3. Testing this using simulated dark matter halos at *z* = 3 showed that we can measure the Hubble parameter at 0.7% precision and the growth rate at 7.3%.

Another strong AtLAST science case revolves around Line-intensity mapping (LIM), which provides an alternative method for studying the large-scale structure of the Universe across cosmic time: it measures 2- and 3-dimensional power spectra from spectral cubes. At sub-mm wavelengths this bridges the optical surveys at *z ≤* 1 and the upcoming radio surveys of the 21-cm HI emission line in the Epoch of Reionisation ( *z ≥* 6). The [CII] and/or CO lines remain bright for 1 *< z <* 6, making them ideal LIM tracers. Current effort are limited by small surveys areas, which AtLAST can resolve as it has a much larger collecting area, large focal plane, and superior site and dish quality. We demonstrated the power of LIM using a CO line to constrain the cosmic expansion history *H*( *z*), i.e the Hubble expansion rate as a function of redshift. This is significantly improved for *z >* 3 when one adds CO LIM to information from Supernovae (SN), galaxy surveys and the Lyman- *α* forest.

Finally, we also presented the science case for mapping several thousand galaxy (proto)clusters at *z* = 1 − 10 with AtLAST, producing a high-redshift counterpart to local large surveys of rich clusters like the well-studied Abell catalogue. The main aims of such a large survey of distant clusters are the formation and evolution of cluster galaxies over cosmic time and the impact of environment on the formation and evolution (possibly environmental) of these galaxies. To make a big leap forward in this emerging research field, we would need a large-format, wide-band, direct-detection spectrometer (based on MKID technology, for example), covering a wide field of *∼*1 square degree and a frequency coverage from 70 to 700 GHz (which could be split over two instruments).

In conclusion, we have shown that AtLAST is able to yield significant progress is a range of research topics focusing on the distant Universe and cosmology, notably the overall high-z galaxy population, the ones located in protoclusters, and the measurement of several cosmological parameters to help constrain cosmological models. This is made possible because of the high angular resolution that a 50-meter aperture brings, its wide spectral coverage with moderately high spectral resolution, and an excellent sensitivity that can be reached over large patches of the mm/sub-mm sky, which is unprecedented.

## Ethics and consent

Ethical approval and consent were not required.

## Data Availability

No data are associated with this article.

## Software availability

The calculations used to derive integration times for this paper were done using the AtLAST sensitivity calculator, a deliverable of Horizon 2020 research project “Towards AtLAST”, and available from this link. All other calculations were derived from previously existing software (code not developed for this paper), details of which can be found in the literature cited.
