# Supplementary material for: Atacama Large Aperture Submillimeter Telescope (AtLAST) science: Surveying the distant Universe
Source: Open Res Eur. 2024 Jun 24;4:122. [Version 1] doi: 10.12688/openreseurope.17445.1 (PMC11472272; doi:10.12688/openreseurope.17445.1)
Supplement: Peer review history

# Reviewer report: Reviewer response for version 1

**DOI:** 10.21956/openreseurope.18852.r44668

**Published:** 2024-10-24

**Authors:** Hideki Umehata

**Affiliations:** 1 Nagoya University, Nagoya, Japan

The AtLAST is a next generation single-dish telescope at submm/mm. The large dish size (50m) and  its characteristic, single-dish, will provides a wide field of view with moderate angular resolution and good sensitivity, offering a new parameter space that have not been covered by ALMA. This paper summarizes prospects for (parts of) science cases with AtLAST. The covered area includes galaxy formation and evolution and cosmology. For each science case, the originality of AtLAST and the necessity is generally well described, presenting some quantities such as mapping speed. I do not find major issues, and hence only leave several minor things.

 Section1

 - p6: ADF22 (Umehata+15) would be worth referring.

 - p6: "AzTEC on JCMT/ASTE" would be worth citing.

 Section2

 - p7: Fujimoto et al. 2023 argued that most of CIB are resolved. Referring the work would be a reasonable option.

 - p11: how to measure metallicity for the AtLAST sample? Adding some prospects would be valuable.

 - p11: It is true that optical spectroscopy is not so powerful. At the same time, recently JWST routinely detect rest-frame optical lines. Referring to JWST, also showcasing its limitation and necessity of AtLAST, would be fair.

 - p13: discussion in Sec.2.1 relies on selection of dark matter halos. Concerns (not critical, though) would be (i) only portion of DM halos host DSFGs, and (ii) conflict with previous assumption: [CII]/IR ratio is not deficit since target range is not super bright. Some follow up statement may be favorable.

 - p15: While [CII] over 1000 deg-2 may be reasonable but the case of CO(1-0) may be more challenging. Adding some comments may be an option (e.g., availability of other transitions?)

 Section3

 - p17: In the section, justification for frequency setup (frequency coverage, R, etc) may be more focused than sensitivity. It may be an option to add some sentences to link previous sensitivity estimates in section2.

 https://arxiv.org/abs/2303.01658

Is the case presented with sufficient detail to be useful for teaching or other practitioners?

Yes

Is the work clearly and accurately presented and does it cite the current literature?

Yes

If applicable, is the statistical analysis and its interpretation appropriate?

Yes

Are all the source data underlying the results available to ensure full reproducibility?

Yes

Are the conclusions drawn adequately supported by the results?

Yes

Is the background of the case’s history and progression described in sufficient detail?

Yes

Reviewer Expertise:

My main field is observational study of galaxy formation and evolution, including proto-clusters. There would be no significant issues in reviewing the draft.

I confirm that I have read this submission and believe that I have an appropriate level of expertise to confirm that it is of an acceptable scientific standard.

## References

[1] : ALMA DEEP FIELD IN SSA22: A CONCENTRATION OF DUSTY STARBURSTS IN Az. *The Astrophysical Journal* .2015;815(1) : 10.1088/2041-8205/815/1/L8 10.1088/2041-8205/815/1/L8

# Reviewer report: Reviewer response for version 1

**DOI:** 10.21956/openreseurope.18852.r44666

**Published:** 2024-10-18

**Authors:** Robert M. Yates, Daniel Smith

**Affiliations:** 1 University of Hertfordshire, Hatfield, UK

This white paper provides an overview of the proposed AtLAST telescope's capabilities to observe the high-redshift (z>1) Universe. In particular, predictions for its ability to provide galaxy continuum and emission-line catalogues, inferences on cosmological

 parameters, line-intensity mapping, and galaxy protocluster surveys are presented.

 The overall science case presented here appears well justified, although it is rather qualitative in places. It is indeed important to probe down to lower star-formation rates (SFRs) in high-z galaxy surveys, as these galaxies are known to contribute significantly to the total cosmic HI and SFR density in simulations. Likewise, the importance of probing dusty star-forming galaxies (DSGs) in particular is made clear. The need for a telescope like AtLAST to do this is also argued convincingly. Therefore, while our expertise does not cover the full range of topics discussed, we are able to approve this article with minor reservations. These reservations are outlined below:

 MAIN POINTS: 

 1) ​ATLAST will clearly do some fantastic science, however we do not agree that the proposed surveys would provide an unbiased view. Would it not be fairer to describe these surveys as having bias which differs from those surveys that have gone before? Rather than focussing on sources identified e.g. on the basis of their star light, AtLAST would identify sources on the basis of their dust content (in the case of

 the continuum surveys) or CO/CII/FSL luminosity (in the case of the spectral survey). There are a number of instances of this in the article  where we think the wording should be modified. E.g. in the abstract when referring to how ATLAST allows the study of the ‘whole population of high-z galaxies’, in the plain language summary (‘unbiased’ – which we would also question whether it is plain language), and in section 3.1 (“a more complete census of the sources in an unbiased wide-field redshift survey”), in section 3.2 (“will yield an unbiased survey”).

 2) The reviewers would like to see more of a comparison with the planned LMT public surveys with TolTEC (e.g. http://toltec.astro.umass.edu/science_legacy_surveys.php). TolTEC has a continuum camera with sub-mm sensitivity and a comparable sized dish to what is proposed for ATLAST, so it would be great to see some quantitative statements about how the example surveys described here compare in detail with what is actually being planned (or indeed, already observed) at a level other than the faster survey speed of ATLAST (e.g. sensitivity, area, resolution).

 3) In figure 4 in the top panel, what is the origin of the redshift gaps in detectability? Is this something that could be improved with greater bandwidth, or is it an atmospheric effect? These features are also perhaps apparent in the bottom panel, which is very difficult to read and understand – are these histograms? Irrespective, a little more scaffolding to help the reader understand e.g. in the caption (many of the figure captions would benefit from being more detailed).

 4) There are several places where qualitative statements are made, e.g. in the first sentence of the Introduction – ‘which results in a fair amount of astrophysical dust and gas in these galaxies’ and again in Section 2.1 where 'a fair fraction' appears. It is

 not clear how the reader show interpret ‘a fair amount’ – presumably this is e.g. not meant to imply that it is evenly shared? Perhaps some quantitative statement would be better. Other similar examples that could be reworded include:

 - Section 2.1 refers to the ‘detection rate of recent ALMA Large Programs’ like ALPINE and REBELS. It would be good to say what that detection rate is in those surveys if this is part of the motivation for [CII] with ATLAST.

 - In section 2.1.1: ‘Interestingly, this continuum survey will contain many Virgo/Coma-like structures’. How many will it contain, and in what way are they Virgo/Coma-like?

 - Section 2.1.2: ‘Such a survey will basically provide multiple line detection for galaxies below SFR* up to z ~ 7’. Rather than ‘basically’ it would be much stronger to provide a quantitative statement about the percentage of galaxies for which multiple line detections will be available; on the basis of the bottom panel of figure 4 (which I admit I have a hard time reading as currently included) this figure would seem to be around 80%?

 - A similar usage of ‘basically’ is in the last paragraph beginning on page 11 – indeed it seems to repeat the previous appearance. Could this sentence be left out?

 ABSTRACT:

 - The term "the high-z galaxy population" should be quantified in the Abstract. Later on, it is defined as z>1, so that should be made clear here too.

 - Clearly, AtLAST will not resolve "all galaxies", but rather will add to the overall inventory of observable galaxies though its ability to detect DSGs down to quite low masses. This should be clarified in the text.

 PLAIN LANGUAGE SUMMARY:

 - "the higher efficiency of galaxies at converting their gas into stars." As we understand it, the star-formation efficiency (SFE) as normally defined is only expected to be slightly higher at high redshift (see e.g. Tacconi+13; Saintonge & Catinella 21). Also, this temporal variation is predominantly an indicator of the change in gas inflow rate with cosmic time, rather than a change in the intrinsic efficiency of converting dense molecular gas into stars. If this is correct, it should be clarified in the text.

 INTRODUCTION:

 - "the star formation rate density in galaxies is highest in the redshift range 1 < z < 3 ... which results in a fair amount of astrophysical dust and gas in these galaxies." We wouldn't say that star formation results in a lot of gas. Rather, it is the other way around. This sentence should probably be re-worded.

 - "Since high-z star forming galaxies" --> "Since high-z dusty star forming galaxies"

 - "Since high-z star forming galaxies are best observed in the (sub-)mm they are often called ‘sub mm galaxies.’" To our understanding, dusty star-forming galaxies are not just a renaming of the population. Rather, sub-millimetre galaxies are a subset of dusty star-forming galaxies (i.e. the ones which were originally a challenge for models to reproduce on account of their very large SFRs). If we should update our understanding, or if something else was meant by the authors, please discuss here.

 - There are problems with the direction of the inverted commas throughout – please fix such that the preceding and consequent inverted commas are ‘leaning’ in opposite directions.

 - Related studies from ALPINE and MORA (e.g. Gruppioni+20; Zavala+21) seem to be missing from the discussion of ALMA surveys here.

 - Fig. 1 caption: "confusion noise at X mJy level". The  X should be replaced by the true value.

 - Please update ‘negative K-correction at this wavelength regime enables galaxies to appear roughly constant in the observed flux densities’ to highlight that it is galaxies *of a particular luminosity*.

 - “The redshift distribution depends on the selection wavelength (e.g. Bethermin et al. 2015)” – it may also be worth mentioning that it also depends on the selected flux density (e.g. there is a nice paper by Smith and collaborators in 2017 that touches on this, which also obtained an effectively complete redshift distribution for SMGs without requiring sub-mm spectroscopy, this is relevant for elsewhere in this work).

 - Remove unnecessary ‘of interferometers’ at the end of the sentence beginning ‘Instead, the interferometers act…”

 - Typo referring to the stellar mass of galaxies in ALPINE/REBELS as 1^{10} in the penultimate paragraph of page 6.

 SECTION 2:

 - ‘as well on galaxies’ --> ‘as well as on galaxies’

 - In the first paragraph of section 2.1, it is a little clunky to talk about 150um in the rest frame in the context of the CIB (which doesn’t have a single rest frame since it is the sum of the emission by all galaxies emitted over all redshifts).

 - "the evolution of the dust properties over cosmic time, which is under intense debate (see e.g. Dayal et al., 2022; Di Cesare et al., 2023; Drew & Casey, 2022; Hirashita & Il’in, 2022; Sommovigo et al., 2022)." We note that there have been other recent simulation-based works in this area recently, e.g. Parente+22; Yates+24; Hough+24.

 - Typo: "I Another approach"

 - "Both approaches are severely biased against the brightest end of the population." We find this statement rather confusing about the MIR- and radio-selected samples, and the lensed population. Please clarify.

 - "Especially [CII] is a very valuable tracer, physically. It ... is a valid tracer of the bulk of the gas mass". More precisely, [CII] traces predominantly the neutral gas mass within galaxies (see e.g. Wolfire+22; Heintz+22). This should be clarified in the text.

 - We would question whether the first sentence of the second paragraph of section 2.1 is correct as currently stated – much is known observationally about normal dusty galaxies, since they are the ones that are readily detected and characterised by e.g. near-infrared surveys (that several of the authors of this work have spent many years working on), and at lower redshifts with e.g. Herschel. Perhaps it is worth clarifying that you are referring to detecting normal star-forming galaxies at high-z in emission at sub-mm wavelengths?

 - The sentence beginning ‘Or the evolution’ seems to be incomplete.

 In section 2.1 the authors talk about how some wavelengths could ‘hit the confusion limit for the longer wavelengths if the survey area is too small’; this seems a rather strange way to phrase this, since the confusion limit is unchanging as a function of survey area or integration time (being a result of the intrinsic resolution of the observatory). Perhaps this is an unfortunate artefact of discussing an example survey of fixed duration, rather than of a sensitivity that is defined on the basis of wishing to achieve particular science goals? The text here should be modified to clarify this issue.

 - "Such a continuum survey allows one to infer two important physical properties of galaxies: their infrared luminosities LIR (and thus SFR obscured) and dust masses (subsequently M_ISM, assuming a given gas-to-dust ratio)". The use of a fixed gas-to-dust ratio here seems overly simplified, given that this ratio (as well as the associated dust-to-metal ratio) is known to vary with e.g metallicity (see e.g. DeVis+19; Yates+24). Although this does not impact the efficacy of the underlying AtLAST observations, the issue should be mentioned here.

 - "in 2" --> "in Figure 2".

 - ​In section 2.1.1, the authors note that the proposed survey needs the best observing conditions for the 350/450um band observations. How does the proposed observing time (1000hr) compare to the typical availability of the necessary conditions during a typical year?

 - It should be made clearer whether the trends shown in figure 2 account for sources of a particular luminosity range (as I might expect for ‘mock observations’ such as those referred to in the caption) or indeed to the more idealised (or perhaps less realistic) scenario of 5 sigma in every band, when even with the benefit of the negative K-correction the signal-to-noise ratio observed is a function of luminosity (and at the other bands a strong function of redshift, too). This would provide additional value beyond the general statements in section 2.1.1 about the constraints on luminosity coming from the shorter FIR wavelengths, and on the dust mass coming from the longer wavelengths (which have of course been known for some time).

 - We were surprised to see the number counts and redshift distribution of normal dust[y] star-forming galaxies as principal deliverables described here – we would have thought that more interesting would be the luminosity function and its cosmic evolution (and that these would be readily obtainable with the surveys proposed here). Aiming for the number counts and redshift distribution (in some cases, already well measured) seems rather low-hanging fruit for such a large outlay of observing time as what is being proposed here.

 - In several places (e.g. at the bottom of page 9) the authors switch from quoting band passes in wavelengths to ‘longer frequencies’ the other side of a full stop. It would be easier to follow if either both frequency and wavelength were quoted in all cases, or perhaps even better if either wavelength or frequency was stuck to throughout.

 - “At lower frequencies we hit the confusion limit” – it would be great if this were updated to not only address the previous remark, but also to be accompanied by e.g. a table quoting the anticipated confusion limit for ATLAST at each wavelength being considered.

 - "using dark matter only" --> "using a dark matter only"

 - The list of references at the end of the paragraph beginning ‘Again using the expected mapping speed’ should be in parenthesis.

 - It seems unnecessary to include ‘which is excellent’ at the end of the penultimate paragraph on page 11; preferable would be to quote the numbers and allow the reader to judge for themselves, unless a more quantitative statement could be provided about how these numbers compare to those expected for other existing/planned surveys.

 - "we apply a selection of halos with masses of 10^12.5 − 10^13.5 solar masses, which yields about 800k halos for the analyses." It should perhaps be mentioned here that the central galaxies of such halos will be very massive for this redshift (z~3). These systems should therefore be quite different from the sub-SFR* galaxies discussed in the preceding sections.

 - Figure 5: It would be helpful to explicitly state (in the main text or at least the caption) that Hf(z) is the "true" value of the Hubble parameter for the simulation used. Thus, H(z)/Hf(z) provides an indication of the uncertainty in measuring the Hubble parameter from DSG clustering.

 - The disagreement between the various models in Fig. 6 is striking. Therefore, it would be good to explicitly state that [CII] data from AtLAST should help constrain these models. If that is indeed the claim here.

 - "Galaxy clusters are the first large structures to form..." The term "large" is relative here (i.e. depends on redshift), so I would recommend stating "Galaxy clusters typically reside in the most overdense regions of the underlying dark matter large-scale structure", or similar, instead.

 - "Clusters grow hierarchically through the merging and accretion of smaller units of galaxy halos, which are dominated by (very) young galaxies displaying intense bursts of star-formation" This description of cluster members is not necessarily true at all redshifts. If the authors are referring specifically to the early/intermediate stages of cluster formation, they should explicitly state so here, and/or use the term protocluster" from the beginning of this section.

 - "Even though ALMA and ATCA allowed this number to increase significantly ... it remains low nonetheless." No actual numbers are provided here. A quantitative assessment of the number of galaxies that have been observed, and should be observed by AtLAST, would be useful here.

 - Figure 9: The model used to generate the mock image in this figure seems rather simple, compared to more recently galaxy evolution modelling over the last ~20 years. Compelling reasons for why the fraction of observable cluster galaxies it predicts would be similar to that obtained from more recent semi-analytic/hydrodynamical simulations would be good here.

 - ‘will loose many’ should be ‘will lose many’

 - ‘the qudrapole’ should be ‘the quadrupole’

 - In section 2.4 it is not clear to us how the galaxy clusters should be seen as the ‘earliest fingerprint of galaxy formation and evolution’ – we have the impression that little is known about the environments in which the earliest galaxies reside (but please do expound upon this if we're wrong).

 - “several hundred detection’s” à “several hundred detections”

 SECTION 3:

 - There are a number of minor typos in this section. Therefore, we recommend the text is reviewed by the authors or journal editors.

 - "One option would be a heterodyne instrument ... however the costs would be exorbitant, so this will not be feasible. Thus, we opt for the MKID bolometer technology which should provide integral field unit spectroscopic capabilities.” Is more information on heterodyne versus MKID bolometer technology presented in an associated AtAST white paper? If so, these works should be cited here. If not, some more detail should be given in this work.

 SECTION 4:

 - ‘dessert’ should be ‘desert’

 - ‘make mayor contributions’ -> ‘make major contributions’

 - ‘one the brightest’ -> ‘one of the brightest’

Is the case presented with sufficient detail to be useful for teaching or other practitioners?

Yes

Is the work clearly and accurately presented and does it cite the current literature?

Partly

If applicable, is the statistical analysis and its interpretation appropriate?

Yes

Are all the source data underlying the results available to ensure full reproducibility?

Yes

Are the conclusions drawn adequately supported by the results?

Partly

Is the background of the case’s history and progression described in sufficient detail?

Yes

Reviewer Expertise:

Galaxy evolution theory & galaxy surveys and (sub-)millimeter astronomy

We confirm that we have read this submission and believe that we have an appropriate level of expertise to confirm that it is of an acceptable scientific standard, however we have significant reservations, as outlined above.

# Reviewer report: Reviewer response for version 1

**DOI:** 10.21956/openreseurope.18852.r43137

**Published:** 2024-10-11

**Authors:** Rodrigo Carvajal

**Affiliations:** 1 Universidade de Lisboa, Lisbon, Lisbon, Portugal

This article presents several case studies, related to high-redshift science, in which the use of the future AtLAST telescope can significantly improve the quality of their results.

 The high projected surveying speed, coupled with wide field, very wide band, and deep observations, are expected to boost the study of large number of high-redshift galaxies, (proto-) galaxy clusters, line intensity mapping (LIM), and fundamental cosmological parameters.

 General Comments:

 While the article is well structured and touches a large amount of technical capabilities that can help several lines of research, there are some parts of it that were not completely clear to this referee and would need further development or better description to fit better in the overall organisation of the article.

 In general, there are sections and paragraphs that clearly show the work of several/different authors. While this is not a problem by itself (it is indeed expected), there is a significant amount of information that is introduced several times throughout the text, increasing the length of the article unnecessarily. Such repetitions include multiple definition of some concepts with different names that might confuse the reader.

 Specific comments:

 1 Introduction

 - First paragraph, last sentence: A clear reference to the first detection/discovery of DSFGs might improve the inclusion of this sentence.

 - Second paragraph, penultimate sentence: Please include a rough value of the angular resolution previous surveys have (orders of magnitude are more than enough).

 - Second paragraph, last sentence: Giving a few more details about these facilities would match the descriptions of the first part of this paragraph.

 - Third paragraph, last sentence: A more explicit description of what frequency range would enable a complete census of dusty galaxies would be a relevant addition. As it is now, the sentence seems somehow vague.

 - Fourth paragraph, first and second sentences: These sentences fit better in the previous paragraph. Thus, this paragraph should start with the third sentence.

 - Fourth paragraph, penultimate sentence: Typo, 1^1 should be 10^1.

 - Fifth paragraph, last sentence and Figure 1: Is it possible to give more details about Figure 1? It is clear that it is meant to compare confusion and detection limits, but a very succinct description of the simulations themselves (i.e. Lagos et al. 2020) might help.

 - Figure 1: Typo, 'limited by confusion noise at X mJy level'. X should be replaced.

 2 Science goals

 2.1 A large homogeneous galaxy survey in the distant Universe

 - Second paragraph, antepenultimate sentence: Please include a reference or a very short description of how the properties of MIR- or radio-selected galaxies have been extrapolated to study less extreme sub-mm galaxies.

 - Second paragraph, penultimate sentence: How analysing the brightest DSFGs (even though some of them are lensed) can help studying fainter systems? Earlier in the text, it has been said that the existence (and study) of very extreme sources is not enough to analyse the bulk of the DSFG population. A short description of such approach could help the reader.

 - Fourth paragraph, last sentence: Please elaborate on why (and how) Euclid, LSST, Roman, and SKA can complement AtLAST observations. Is it only because of the large number of sources available to observe with AtLAST?

 -Sixth paragraph, first sentence (parenthesis): It is clear that knowing, in advance, the positions of high-redshift sources can improve the performance of AtLAST. But it is not completely understood why other science cases could be boosted by imaging surveys but galaxy surveys not. Please elaborate.

 2.1.1 A wide continuum survey

 - First paragraph: Some sentences in this paragraph contain repeated information. For instance, the description/reference of Figure 2. Please re-format this paragraph to avoid redundancies.

 - Third paragraph, first sentence: What do the authors refer to with 'classic star formation rate'? It is not completely clear if this is related to typical values or something else. Please expand.

 - Fourth paragraph, second sentence: This sentence highlights an innovative capability brought by AtLAST. Expanding its description might help proving the excellent potential AtLAST has.

 - Fourth paragraph, fifth sentence (parenthesis): Typo: number of bands is missing.

 - Fourth paragraph, sixth sentence: A reference for the current number of available galaxies with very good L_IR and M_dust accuracy might help the reader to understand the improvement brought by AtLAST (or at least the order of magnitude).

 - Fourth paragraph, penultimate sentence: The presence of Virgo/Coma-like structures in the surveys is not completely described here. The addition of a sentence on why/how this is relevant can help the reader.

 2.1.2 A deep "blind" spectroscopic survey

 - Third paragraph, second sentence (parenthesis) and Figure 4: The way in which lines are labelled is somehow confusing. Improving the legend or the figure caption would help the reader clearly identify each line with a transition.

 - Is AtLAST the only instrument/survey planned to obtain similar results? It is clear that existing facilities cannot reach its performance level, but a mention to future surveys (if any) can help highlighting the capabilities of AtLAST.

 2.2 Constraining cosmological parameters via BAO and clustering

 - Second paragraph, last sentence: An explicit mention to the instrument used to detect these lines (ALMA) is missing.

 - Third paragraph: The works by Wilkinson et al. (2017) and Amvrosiadis et al, (2019) have already used sub-mm observations to obtain their conclusions. Thus, the pertinence of including them in this paragraph is not clear.

 - Fourth paragraph, third sentence: What is the smallest simulation box that might still be useful for this exercise? Please include a very succinct description of why selecting realisations from DARK QUEST.

 - Seventh paragraph: Is it possible to include a very short description/discussion of what treatment should AtLAST data have in order to be used for these cosmological calculations. In other words, does the improvement AtLAST brings only come from the large number of sources?

 2.3 Line-intensity mapping (tomography)

 - First paragraph, first sentence: A reference for a general description of LIM would be useful.

 - Figure 5: How is the simulated data shown in this figure related to what AtLAST will observe? Is it possible to describe the direction in which the curves in this figure will move to?

 - Figure 6: Is it possible to include in this figure (or in the caption or main text) a description of the range of values AtLAST is expected to cover?

 2.3.2 Current observations: lack of constraints

 - First paragraph, last sentence: Will any other facility help improving/constraining LIM signal? Even though at smaller scales, what other efforts can help in this direction?

 - Third paragraph, last sentence (Figure 7): The impact of AtLAST is not completely clear from Figure 7 only. More comments are needed in order to assess the contribution of AtLAST (e.g. how much will the curves distribution will be shrunk).

 2.4 Surveying cluster galaxies in the distant Universe

 - Figure 8: Figure is not completely clear at showing what the caption says. Please assess the pertinency of presenting this figure as related to the main text.

 2.4.1 A systematic mapping survey of distant cluster galaxies

 -  First paragraph, first sentence: What is the reason for current samples of galaxies not being able to sample full infall regions? A better explanation of the reasons can help the reader.

 - Second paragraph, last sentence: Authors mention that current sub-mm facilities cannot *practically* cover areas up to one square degree with adequate sensitivity and survey speed. Is it possible to include example(s) of how is this possible with current facilities?

 2.4.2 The way forward

 - Figure 9: From reading the caption and main text, it is not completely clear whether this is a mock AtLAST observation or not. Please include more explicit information about the origin of such simulation.

 3 Technical justification

 3.1 AtLAST as a sub-mm redshift machine

 - Fourth paragraph, last sentence: In which way a way band IFU would be highly efficient at z > 2? Please include more details about this exercise. Additionally, this sentence should be more strongly highlighted. If possible to reach such broad redshift range, AtLAST by itself would be able to create a CO ladder of its own to reach very high z values.

 - Fifth paragraph, last sentence: What is the process to bin high-resolution spectra in AtLAST? Can this be done from the side of AtLAST (at the moment of observation) or is the final user who should do it by themself?

 3.2 Surveying proto-cluster galaxies

 - Second paragraph, third sentence: CO SLED has been previously introduced in the article as CO ladder. Please check consistency in the text.

 - Third paragraph, first sentence: Cosmic variance has not mentioned before. How relevant is it for AtLAST observations. Given its large field of view and deep projected observations, what is its role in cluster observations? If needed, a proper introduction of the subject can be included earlier in the text.

 - Third paragraph, second sentence and list: Are the requirements listed here only for galaxy cluster science? If so, similar lists should be produced for the remaining science cases.

 4 Summary and conclusions

 - Third paragraph, first sentence: Auto-correlation function has been mentioned previously in the text, but as two-point correlation function. Please keep the naming consistent.

 - Third paragraph: This paragraph raises the question of how useful will AtLAST be for blind (DSFGs) surveys. Such topic is not fully described in the text. A comment on that could be incorporated.

 - Eight paragraph, first sentence: Abell catalogue was mentioned (in the main text) only as an example of a local catalogue to be replicated at high-redshift values. No proper (or direct) estimations were made for AtLAST. Please check consistency of the text.

Is the case presented with sufficient detail to be useful for teaching or other practitioners?

Yes

Is the work clearly and accurately presented and does it cite the current literature?

Yes

If applicable, is the statistical analysis and its interpretation appropriate?

Not applicable

Are all the source data underlying the results available to ensure full reproducibility?

Partly

Are the conclusions drawn adequately supported by the results?

Yes

Is the background of the case’s history and progression described in sufficient detail?

Yes

Reviewer Expertise:

Radio Galaxies, Machine-assisted analysis, AGN, Lyman-Break Galaxies.

I confirm that I have read this submission and believe that I have an appropriate level of expertise to confirm that it is of an acceptable scientific standard.

## References

[1] : The SCUBA-2 Cosmology Legacy Survey: the clustering of submillimetre galaxies in the UKIDSS UDS field. *Monthly Notices of the Royal Astronomical Society* .2017;464(2) : 10.1093/mnras/stw2405 1380-1392 10.1093/mnras/stw2405

[2] : Herschel-ATLAS : the spatial clustering of low- and high-redshift submillimetre galaxies. *Monthly Notices of the Royal Astronomical Society* .2019;483(4) : 10.1093/mnras/sty3013 4649-4664 10.1093/mnras/sty3013

[3] : Physical properties and evolution of (sub-)millimetre-selected galaxies in the galaxy formation simulationshark. *Monthly Notices of the Royal Astronomical Society* .2020;499(2) : 10.1093/mnras/staa2861 1948-1971 10.1093/mnras/staa2861

# Reviewer report: Reviewer response for version 1

**DOI:** 10.21956/openreseurope.18852.r44671

**Published:** 2024-10-10

**Authors:** Fabrizio Gentile

**Affiliations:** 1 University of Bologna, Bologna, Italy

In this article, the authors present a case study of the future Atacama Large Aperture Submillimeter Telescope (AtLAST) and some scientific goals that could be achieved with the construction of this new facility. These focus on the collection of large samples of high-z dusty star-forming galaxies (DSFGs), on the constraints on cosmological parameters through a clustering analysis, on the study of the large-scale structure through line-intensity mapping, and on the analysis of high-z cluster galaxies.

 I reviewed the proposed case study and, in my opinion, it clearly presents the potential of the AtLAST telescope in the proposed scientific goals. Nevertheless, I have some (partly major) comments that the authors could address before the article is indexed. Since some of them could affect the feasibility of the proposed science goals, more comments could arise once these are addressed.

**Mayor comments:**

1. My main comment on the current version of the article is that it does not present (in the introduction or in an initial section) the AtLAST telescope capabilities in a quantitative way. In my opinion, a brief summary would help the readers assess the feasibility of some of the studies proposed in the following sections. As an example, most of the scientific goals about DSFGs presented in Section 2.1 would be hard to achieve if the multi-wavelength counterparts of the (sub)mm sources are not easily identified (e.g. for the estimation of the photo-z). Stating the spatial resolution in the (sub)mm band mentioned in the paper would be particularly useful to strengthen these scientific cases. Similarly, the bandwidth of the observing bands (now mentioned in Section 3.2) would be helpful to assess the feasibility of the redshift estimation through the detection of emission lines presented in Sec 2.1.2.
2. Section 2.1.1: Related to the first comment, it is not clear to me if, in the fitting of the mock observations with the blackbody function, the redshift is assumed to be known without uncertainty. Is this a realistic assumption? Again, I think that this point is strongly related to the ability to constrain the redshift of the multi-wavelength counterpart of each source
3. Section 2.1.1, third paragraph: I think the expression “and classical scaling relations” could be hard to understand for a non-expert reader. Can the authors spend a few lines presenting the used relations?

**Minor comments:**

1. The authors could make the description of the other surveys presented in the introduction more quantitative. Expressions like “shallow” (referred to the SPT), “several deg2” (referred to LABOCA and SCUBA-2) should be quantified by giving at least one order of magnitude for the sensitivity and covered area. A similar comment holds for the FIR facilities mentioned in the following paragraph
2. The authors could provide the updated citation for the extended-MORA survey presented in the introduction (Long+24; arXiv:2408.14546)
3. When the authors present the spectral-line surveys in the introduction, the covered area is included only for some of them. They could consider adding this information to the other surveys mentioned in the paragraph
4. Can the authors present some examples (with references) of the “few exceptions” mentioned in the same paragraph about the spectroscopic instruments on single-dish telescopes?
5. Can the authors clarify what they mean by “the less extreme population of normal DSFGs” presented in the second paragraph of section 2.1? Maybe a more quantitative description of the (sub)mm flux or the physical properties could make the expression more clear
6. Can the authors provide some references for the MIR- and radio-selected galaxies with FIR/(sub)mm emission in the third paragraph of Section 2.1?
7. Can the authors describe the range of parameters employed to model the SEDs in Section 2.1.1?

Typos (not including those already reported by the first referee):

1. Introduction, fourth paragraph: “1^10” -> 10^10 Msun
2. Section 2.1, first paragraph: “the contribution … are” -> “the contribution … is”
3. Section 2.1, fifth paragraph: “efficiently:” -> “efficiently”
4. The citation format in the second paragraph of Section 2.1.2 is wrong (in latex \citet instead of \citep)
5. Section 2.1.2, first paragraph: “CII” -> “[CII]”
6. Section 2.1.2, third paragraph: 610 and 51 should be subscripts

Is the case presented with sufficient detail to be useful for teaching or other practitioners?

Partly

Is the work clearly and accurately presented and does it cite the current literature?

Partly

If applicable, is the statistical analysis and its interpretation appropriate?

Partly

Are all the source data underlying the results available to ensure full reproducibility?

Yes

Are the conclusions drawn adequately supported by the results?

Partly

Is the background of the case’s history and progression described in sufficient detail?

Yes

Reviewer Expertise:

Formation and evolution of galaxies; Dusty star-forming galaxies at high-z; (Sub)mm astronomy

I confirm that I have read this submission and believe that I have an appropriate level of expertise to confirm that it is of an acceptable scientific standard, however I have significant reservations, as outlined above.

# Reviewer report: Reviewer response for version 1

**DOI:** 10.21956/openreseurope.18852.r43139

**Published:** 2024-09-18

**Authors:** Nickolas Kokron

**Affiliations:** 1 Princeton University, Princeton, New Jersey, USA

This case study presents a white paper arguing for the construction of the Atacama Large Aperture Submillimiter Telescope (AtLAST), a 50m sub-mm highly multiplexed facility operating in the Atacama Desert. An overview is presented of the science that can be delivered by such an instrument, anchored in a comprehensive overview of the literature surrounding sub-mm science and line intensity mapping. This white paper presents four science cases focused on 

 1. A deep and a wide survey of the sub-mm Universe from z=0-8 with the intent of studying the evolution of the global cosmic star formation rate, IR luminosity and dust properties of galaxies.

 2. A spectroscopic survey of [CII] emitters at z~3 whose 3D clustering measurements will provide measurements of the expansion and growth of structures in the Universe. 

 3. A Line-intensity mapping (LIM) survey which will measure highly redshifted CO rotational transitions as well as [CII] emission from unresolved sources over large volumes. 

 4. A high redshift galaxy cluster survey aimed at imaging their cold gas. 

 The AtLAST telescope would be uniquely suited to carry out many of these scientific analyses and this proposal comes at a time in which many smaller-scale LIM pathfinders are currently vying for a first detection of the kinds of signals AtLAST will measure at scale. 

 I believe this white paper is an appropriate and timely case study but there are a few elements which I would like to see addressed before its indexing. I lay these out below: 

 Major: 

 The clustering survey proposed in section 2.2 promises to deliver significant results but I am unsure as to whether the set-up employed for this forecast is achievable by AtLAST. Some of the key points laid out are below: 

 1. The forecast in section 2.2 of a 0.7% measurement of the Hubble constant at z=3 is entirely due to the volume of the simulation at 8 (Gpc/h)^3 used to measure halo clustering. Given a survey area of 1000 square degrees, how does AtLAST plan to achieve these volumes? From a back of the envelope calculation the volume of the shell from 2.5 < z < 3.5 at 1000sqdeg is of 4 (Gpc/h)^3. 

 2. Assuming a volume is surveyed corresponding to 8 (Gpc/h)^3 (or a more realistic volume for the survey is provided), how certain are we that the clustering of these [CII] emitters corresponds to the clustering of halos in the mass bin of 10^12.5 - 10^13.5? Emission-line galaxies with OII features at z~1.5 are probing lower masses of 10^12 and this corresponds to a lower bias, which would again lower the SNR of the clustering measurement.

 Figure 6 illustrates the large variation in predictions of the [CII] LIM power spectrum given current astrophysical uncertainties. Which of these models will AtLAST be able to constrain or rule out given the instrument specifications and the proposed LIM survey in section 2.3? It is also not clear to me that AtLAST's surveys of CO / CII could achieve constraints on the expansion history as shown in Fig 7, given what the white paper has laid out.

 In general, the lack of clear quantitative results delivered by these proposed surveys, coupled with concerns reported in the spectroscopic forecast for section 2.2, makes me wary that similar issues could be present in sections of this work not as close to my own expertise. 

 Minor:

 1. It's not immediately clear to me that the caption in Fig 2. lines up with the content of the Figure. It says the mean L_IR and M_dust values are shown in the top panel and the dispersion in the bottom panel, but their y-axes labels claim that the top panel is related solely to L_IR values and the bottom panel is related solely to M_dust panels. Could the authors clarify if the caption correctly describes the figure? 

 2. The authors refer to the "expected mapping speed of AtLAST" already in section 2.1.1 to arrive at a flux sensitivity for their survey. I believe this number is derived from the AtLAST sensitivity calculator mentioned in the "Software availability" section. To ensure this white paper is self-contained, it might be beneficial to give a brief overview of how this calculator works and what this number for the mapping speed depends on. 

 3. Figure 9 shows a mock image of CO flux around a protocluster. Could the authors highlight the spatial extent of the protocluster in this image? It's unclear from the figure where it should be located and what else in the map is, e.g., cosmic web. 

 Very minor:

 1. In Figure 1 the value for the confusion noise of the ACT map is left as an "X" without the real value included. 

 2. The usage of "till" in the second paragraph following Section 2.1 is colloquial and should be replaced with "until". In this same paragraph there is a typo -- "I Another". 

 3. The paragraph immediately preceding section 2.2 has the word "loose" instead of "lose". 

 4. In section 2.2 there is a typo: "quadrapole"

 5. Two paragraphs before section 2.4.2 there is a typo: " detection's " should be "detections". 

 6. Two typos in the second paragraph of section 4: "time" -> "times" and "mayor" -> "major. 

 7. Typo in the final paragraph "is a range" -> "in a range".

Is the case presented with sufficient detail to be useful for teaching or other practitioners?

No

Is the work clearly and accurately presented and does it cite the current literature?

Partly

If applicable, is the statistical analysis and its interpretation appropriate?

Partly

Are all the source data underlying the results available to ensure full reproducibility?

Yes

Are the conclusions drawn adequately supported by the results?

Partly

Is the background of the case’s history and progression described in sufficient detail?

Yes

Reviewer Expertise:

Large-scale structure cosmology, galaxy surveys, line-intensity mapping

I confirm that I have read this submission and believe that I have an appropriate level of expertise to confirm that it is of an acceptable scientific standard, however I have significant reservations, as outlined above.
